# Comparison of degree and modified reverse degree descriptors incorporating entropy and graph energy analysis for graphene allotropes

**DOI:** 10.3389/fchem.2026.1835238

**Published:** 2026-10-22

**Authors:** M. Regina Celine, A. Berin Greeni

**Affiliations:** School of Advanced Sciences, Vellore Institute of Technology, Chennai, India

**Keywords:** bond-wise entropy, graph energy, graphene allotropes, modified reverse degree indices, regression modeling

## Abstract

Graphene allotropes are carbon-based materials with distinctive structural variations that result in diverse electronic, mechanical, and energy applications. Topological descriptors are graph-theoretical metrics widely used to assess physicochemical characteristics of organic metals based on their molecular structure. In this paper, we employ a modified reverse degree-based methodology and analyze the structural complexity using entropy measures. Furthermore, we also calculated the graph energy and developed a regression model, which was compared using modified reverse degree and normal degree-based indices to demonstrate the efficiency of the methodology and provide more comprehensive comprehension into the structural behavior.

## 1 Introduction

Graphene is the fundamental structural layer of graphite, consisting of a one-atom-thick sheet of carbon atoms arranged in a hexagonal lattice (Enyashin and Ivanovskii, 2011). Graphene, which has C-C bonds of approximately 1.42 Å, exhibits exceptional mechanical strength, electrical and thermal conductivity, transparency, and lightness. Graphene is a vital substance for applications in electronics, energy storage, and filtration technologies. However, its zero band gap, limited chemical reactivity, and structural rigidity mean certain applications are restricted, which has prompted the development of novel graphene-based architectures like PHOTH-graphene, S-graphene, and PH-graphene. PHOTH-graphene (PHOTH-G) is a recently developed two-dimensional carbon allotrope composed of carbon atoms arranged in fused four-, five-, six-, seven-, and eight-membered carbon rings, unlike graphene’s purely hexagonal six-atom ring structure (Santos et al., 2024). It contains ten atoms per primitive cell and crystallizes in an orthorhombic lattice with space group P1 , with C-C bond lengths ranging from 0.1410 nm to 0.1435 nm. The armchair and zigzag directions align with the six-membered rings. *Ab initio* molecular dynamics (AIMD) simulations confirm that PHOTH-G demonstrating exceptional mechanical robustness and thermal stability due to its unique ring arrangement, offers better structural flexibility and tunable properties than pristine graphene (Peng et al., 2024).

Sulphur-doped graphene (S-G) is a non-hexagonal graphene-like carbon allotrope that has been thoroughly investigated using Density Functional Theory (DFT) and tight-binding approaches (Xu et al., 2014). There are two Dirac cones in its electronic band structure, which shows that it has mechanical stability; its crystallographic structure is based on a rectangular lattice with eight carbon atoms per unit cell. In addition, S-G can form zigzag-type tubular structures and shows both metallic and semiconducting characteristics, with the electronic properties of its nanoribbon configurations being strongly dependent on the edge geometry (Chegel, 2023). Pentahexoctite-graphene (PH-G) is a two-dimensional graphene that resembles a carbon allotrope and is formed of a periodic arrangement of five-, six-, and eight-membered carbon rings with $sp^{2}$ hybridisation (Brandao et al., 2022). The structure forms a fully planar configuration and exhibits dynamic stablility, as confirmed by the absence of imaginary phonon frequencies in its phonon dispersion spectrum. Owing to its distinct non-hexagonal ring structure, PH-G displays metallic electronic behavior along with direction-dependent anisotropic band dispersion close to the Fermi level. This implies strong anisotropic charge transport characteristics and highlights its potential for advanced nanoelectronics applications (Yogalakshmi and Easwaramoorthy, 2024).

Recent developments in non-classical graphene structures, such as PHOTH-G, PH-G, and S-G, have extended research beyond purely hexagonal lattices through the integration of multiple polygonal faces. These alteration induce lattice distortion, enabling tunable electronic and mechanical properties. Graph-theoretical methods in mathematical chemistry allow the analysis of these structures, using topological descriptors in QSPR and QSAR analysis to relate structure with material behavior. Furthermore, machine-learning models based on graph invariants facilitate effective prediction and screening related to novel graphene materials. Ultimately, graph-theoretical approaches provide a robust framework for investigating molecular structures, their characteristics, and chemical reactivity. Topological descriptors, including hybrid topological descriptors, are essential tools because they generate numerical representations of molecular connectivity that account for the arrangement of atoms and bonds. The development of distance-based descriptors, degree-based descriptors, and hybrid descriptors paved the way for the study of molecular topology to a large extent (Arockiaraj et al., 2023a; Gutman and Trinajstić, 1972; Raza et al., 2023; Wiener, 1947). These descriptors play a crucial role in analyzing nanomaterials, forecasting drug properties, and modeling networks by utilizing structural and entropy-related particulars (Arockiaraj et al., 2024a; Zha et al., 2022; Zhang et al., 2024). In this study, we implement modified reverse degree-based descriptors with a parameter that permits degree sequence variation in the graph. This method strengthens the performance of topological and hybrid topological descriptors, optimizing statistical modeling and predictive analysis for molecular datasets (Arockiaraj et al., 2023a).

Shannon introduced the structural entropy to measure the degree of uncertainty or unpredictability within a dataset (Arockiaraj et al., 2024b; Shannon, 1948). It refers to the many possible configurations of atoms and molecules in a system for better structural disorder with higher entropy values. The chemical structure entropy is a beneficial metric for assessing molecular stability and structural characteristics (Dehmer, 2008). In graph theory, graph entropy measures relate to the probability distributions of the graph components, such as edges and vertices, providing a systematic framework for analyzing complex graph structures. Such methods are particularly beneficial in predicting the graph energies of graphene frameworks by combining graph-theoretical and statistical analyses. Recent studies have enhanced the use of modified reverse degree topological descriptors combined with entropy measures for characterizing and predicting molecular and material properties. The descriptors integrate topological and theoretic information for improving the accuracy of graph energy, spectral, and physicochemical property predictions (Arockiaraj et al., 2024c; Greeni et al., 2024; Kalaam et al., 2024; Kalaam and Greeni, 2024; Kalaam and Greeni, 2025; Roy et al., 2025). While computational approaches investigated algorithmic optimizations in entropy evaluation for MOFs (Waheed et al., 2024). The application of modified reverse degree-based descriptors for graphenes, the development of graph energy prediction models, and the assessment of entropy measures through bond-wise comparative analysis.

The overall workflow of the proposed methodology is shown in Figure 1 to provide a clear overview of the computational procedure used in this study. The workflow summarizes the entire computational pipeline starting from the construction of the graphene molecular graph structures to the computation of the proposed modified reverse degree-based topological descriptors, entropy analysis, graph energy calculation, development of linear regression-based QSPR models, prediction of spectral properties and model validation using statistical performance measures and leave-one-out cross-validation (LOOCV). Overall, the combined application of graph-theoretical and statistical methodologies provides a reliable framework for QSPR modeling and offers potential for the analysis and design of graphene-based materials and related molecular structures.

**FIGURE 1:**
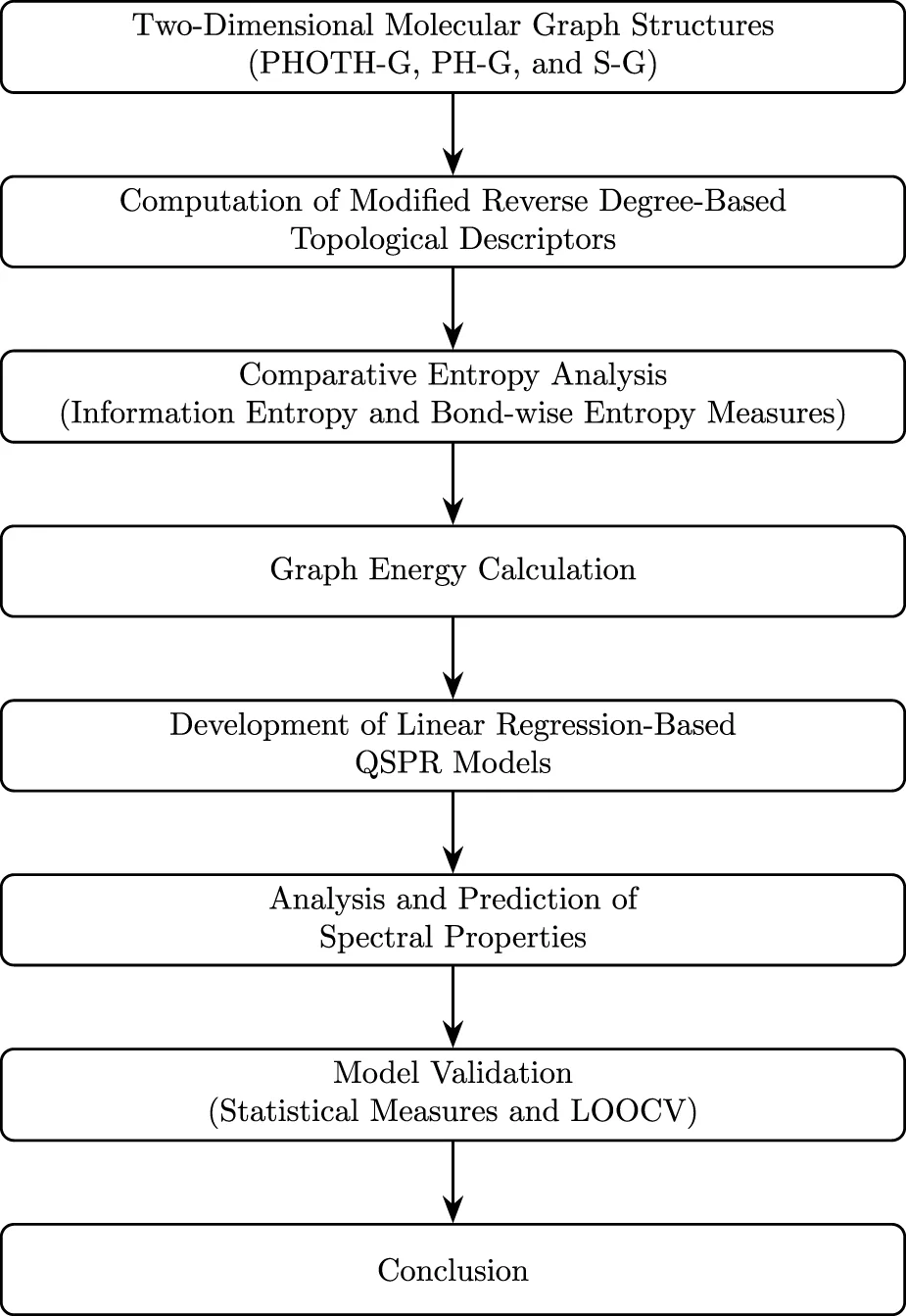
Workflow of the proposed computational methodology for graphene frameworks.

## 2 Methodology

In this study, graphene nanosheets are represented as a two-dimensional molecular graph structure G . The notations |V(G)| and |E(G)| denote the number of vertices (order of G ) and edges (size of G ) in the graph G , respectively. For a given vertex a , the number of vertices adjacent to the vertex a is defined as the degree $d_{a}$ . In the graph G , the maximum degree is given by Δ(G) . In recent years, the modified reverse degree-based approach has received significant attention (Arockiaraj et al., 2023b; Ediz, 2015). It is denoted as $\left(M_{k}R\left(d_{a}\right)\right)$ by introducing a variable parameter k with k≥1 and defined as

$$M_{k}R\left(d_{a}\right)=\left\{\begin{array}{cc} \Delta \left(G\right)-d_{a}+k & :\;k\leq d_{a}\\ \Delta \left(G\right)-d_{a}+k\left(mod\;\Delta \left(G\right)\right) & :\;k>d_{a} \end{array}\right.$$

Therefore, the following expression represents the general form of the topological descriptors (TD) for the modified reverse degree characterization of the graphene framework.

$$M_{k}RTD\left(G\right)=\underset{ab\in E\left(G\right)}{\sum }M_{k}RTD\left(d_{a},d_{b}\right)=\underset{ab\in E\left(G\right)}{\sum }TD\left(M_{k}R\left(d_{a}\right),M_{k}R\left(d_{b}\right)\right)$$

The topological descriptors function denotes $TD\left(M_{k}R\left(d_{a}\right),M_{k}R\left(d_{b}\right)\right)$ ensuring symmetrical mutuality as stated below.

$$TD\left(M_{k}R\left(d_{a}\right),M_{k}R\left(d_{b}\right)\right)=TD\left(M_{k}R\left(d_{a}\right),M_{k}R\left(d_{b}\right)\right)$$

Let the set of edges G be partitioned into equivalence classes $E\left(G\right)=E_{1}\cup E_{2}\cup \cdots \cup E_{n}$ , where each class $E_{i}$ consists of edges having identical modified degree parameters at their end vertices. For any edge $ab\in E_{i}$ , the modified reverse degree-based topological descriptors corresponding to the class $E_{i}$ are defined by,

$$M_{k}RTD\left(E_{i}\right)=|E_{i}|\cdot TD\left(M_{k}R\left(d_{a}\right),M_{k}R\left(d_{b}\right)\right)$$

Therefore, the sum of the contributions from each class $E_{i}$ determines the overall modified reverse degree descriptors for the graph G .

$$M_{k}RTD\left(G\right)=\overset{n}{\underset{i=1}{\sum }}|E_{i}|\cdot TD\left(M_{k}R\left(d_{a}\right),M_{k}R\left(d_{b}\right)\right)$$ (1)

Gutman et al. (1975) first introduced the concept of degree-based topological indices. Later, this concept known as the First and Second Zagreb indices, which characterize molecular branching. These indices became fundamental descriptors in QSPR and QSAR studies due to their strong relationship with molecular properties. Later, the Forgotten Index was revisited by Furtula and Gutman (2015), and the Geometric-Arithmetic index by Vukičević (Gutman et al., 1975; Furtula and Gutman, 2015; Vukičević and Furtula, 2009). The concept of hybrid topological descriptors was introduces by Arockiaraj et al. (2023) by combining the multipe topological indices to predict molecular properties. The main objective of this concept to enchance the accuracy and reliability of predicting molecular properties (Arockiaraj et al., 2023a). Table 1 provides an overview of the topological function for degree and modified reverse degree that is utilized in this study, as defined by Equation 1.

**TABLE 1:** Topological function for degree and modified reverse degree.

TD	Degree form	Modified reverse degree form
Modified first Zagreb $\left(M_{k}RM_{1}\right)$	$d_{a}+d_{b}$	$M_{k}R\left(d_{a}\right)+M_{k}R\left(d_{b}\right)$
Modified second Zagreb $\left(M_{k}RM_{2}\right)$	$d_{a}\cdot d_{b}$	$M_{k}R\left(d_{a}\right)\cdot M_{k}R\left(d_{b}\right)$
Modified forgotten $\left(M_{k}RF\right)$	${\left(d_{a}\right)}^{2}+{\left(d_{b}\right)}^{2}$	${\left(M_{k}R\left(d_{a}\right)\right)}^{2}+{\left(M_{k}R\left(d_{b}\right)\right)}^{2}$
Modified Geometric-Arithmetic $\left(M_{k}RGA\right)$	$\frac{2\sqrt{d_{a}\cdot d_{b}}}{d_{a}+d_{b}}$	$\frac{2\sqrt{M_{k}R\left(d_{a}\right)\cdot M_{k}R\left(d_{b}\right)}}{M_{k}R\left(d_{a}\right)+M_{k}R\left(d_{b}\right)}$
Modified bi-Zagreb $\left(M_{k}RBM\right)$	$d_{a}+d_{b}+d_{a}\cdot d_{b}$	$M_{k}R\left(d_{a}\right)+M_{k}R\left(d_{b}\right)+M_{k}R\left(d_{a}\right)\cdot M_{k}R\left(d_{b}\right)$
Modified tri-Zagreb $\left(M_{k}RTM\right)$	${\left(d_{a}\right)}^{2}+{\left(d_{b}\right)}^{2}+d_{a}\cdot d_{b}$	${\left(M_{k}R\left(d_{a}\right)\right)}^{2}+{\left(M_{k}R\left(d_{b}\right)\right)}^{2}+M_{k}R\left(d_{a}\right)\cdot M_{k}R\left(d_{b}\right)$
Modified harmonic-geometric $\left(M_{k}RHG\right)$	$\frac{2}{\sqrt{d_{a}d_{b}}\;\left(d_{a}+d_{b}\right)}$	$\frac{2}{\sqrt{M_{k}R\left(d_{a}\right)\;M_{k}R\left(d_{b}\right)}\;\left(M_{k}R\left(d_{a}\right)+M_{k}R\left(d_{b}\right)\right)}$
Modified harmonic-tri Zagreb $\left(M_{k}RHTM\right)$	$\frac{2}{\left({\left(d_{a}\right)}^{2}+{\left(d_{b}\right)}^{2}+d_{a}\cdot d_{b}\right)\left(d_{a}+d_{a}\right)}$	$\frac{2}{\left({\left(M_{k}R\left(d_{a}\right)\right)}^{2}+{\left(M_{k}R\left(d_{b}\right)\right)}^{2}+M_{k}R\left(d_{a}\right)\cdot M_{k}R\left(d_{b}\right)\right)\;\left(M_{k}R\left(d_{a}\right)+M_{k}R\left(d_{b}\right)\right)}$
Modified harmonic-bi Zagreb $\left(M_{k}RHBM\right)$	$\frac{2}{\left(d_{a}+d_{b}+d_{a}\cdot d_{b}\right)\left(d_{a}+d_{b}\right)}$	$\frac{2}{\left(M_{k}R\left(d_{a}\right)+M_{k}R\left(d_{b}\right)+M_{k}R\left(d_{a}\right)\cdot M_{k}R\left(d_{b}\right)\right)\;\left(M_{k}R\left(d_{a}\right)+M_{k}R\left(d_{b}\right)\right)}$
Modified bi-Zagreb-geometric $\left(M_{k}RBMG\right)$	$\frac{\left(d_{a}+d_{b}+d_{a}\cdot d_{b}\right)}{\sqrt{d_{a}\cdot d_{b}}}$	$\frac{M_{k}R\left(d_{a}\right)+M_{k}R\left(d_{b}\right)+M_{k}R\left(d_{a}\right)\cdot M_{k}R\left(d_{b}\right)}{\sqrt{M_{k}R\left(d_{a}\right)\cdot M_{k}R\left(d_{b}\right)}}$
Modified bi-Zagreb-harmonic $\left(M_{k}RBMH\right)$	$\frac{\left(d_{a}+d_{b}+d_{a}\cdot d_{b}\right)\cdot \left(d_{a}+d_{b}\right)}{2}$	$\frac{\left(M_{k}R\left(d_{a}\right)+M_{k}R\left(d_{b}\right)+M_{k}R\left(d_{a}\right)\cdot M_{k}R\left(d_{b}\right)\right).\left(M_{k}R\left(d_{a}\right)+M_{k}R\left(d_{b}\right)\right)}{2}$

## 3 Computation of modified reverse degree descriptors

Graphene frameworks are denoted as PHOTH-G (m,n) , S-G (m,n) , and PH-G (m,n) respectively where m and n represent the number of unit cells along the rows and columns, respectively. These graphs whose dimensions (5, 5) are illustrated in Figure 2. The corresponding graph representations possess the following structure: for PHOTH-G (m,n) , |V(G)|=10mn and |E(G)|=15mn−3m−n ; for S-G (m,n) , |V(G)|=8mn and; |E(G)|=12mn−2m−n and for PH-G (m,n) , |V(G)|=8mn and |E(G)|=12mn−2n−m . It is observed that all these graphene frameworks have a maximum vertex degree of 3.

$$M_{1}R\left(d_{a}\right)=\left\{\begin{array}{cc} 2 & :\;d_{a}=2\\ 1 & :\;d_{a}=3 \end{array}\right.$$

$$M_{2}R\left(d_{a}\right)=\left\{\begin{array}{cc} 3 & :\;d_{a}=2\\ 2 & :\;d_{a}=3 \end{array}\right.$$

$$M_{3}R\left(d_{a}\right)=\left\{\begin{array}{cc} 1 & :\;d_{a}=2\\ 3 & :\;d_{a}=3 \end{array}\right.$$

**FIGURE 2:**
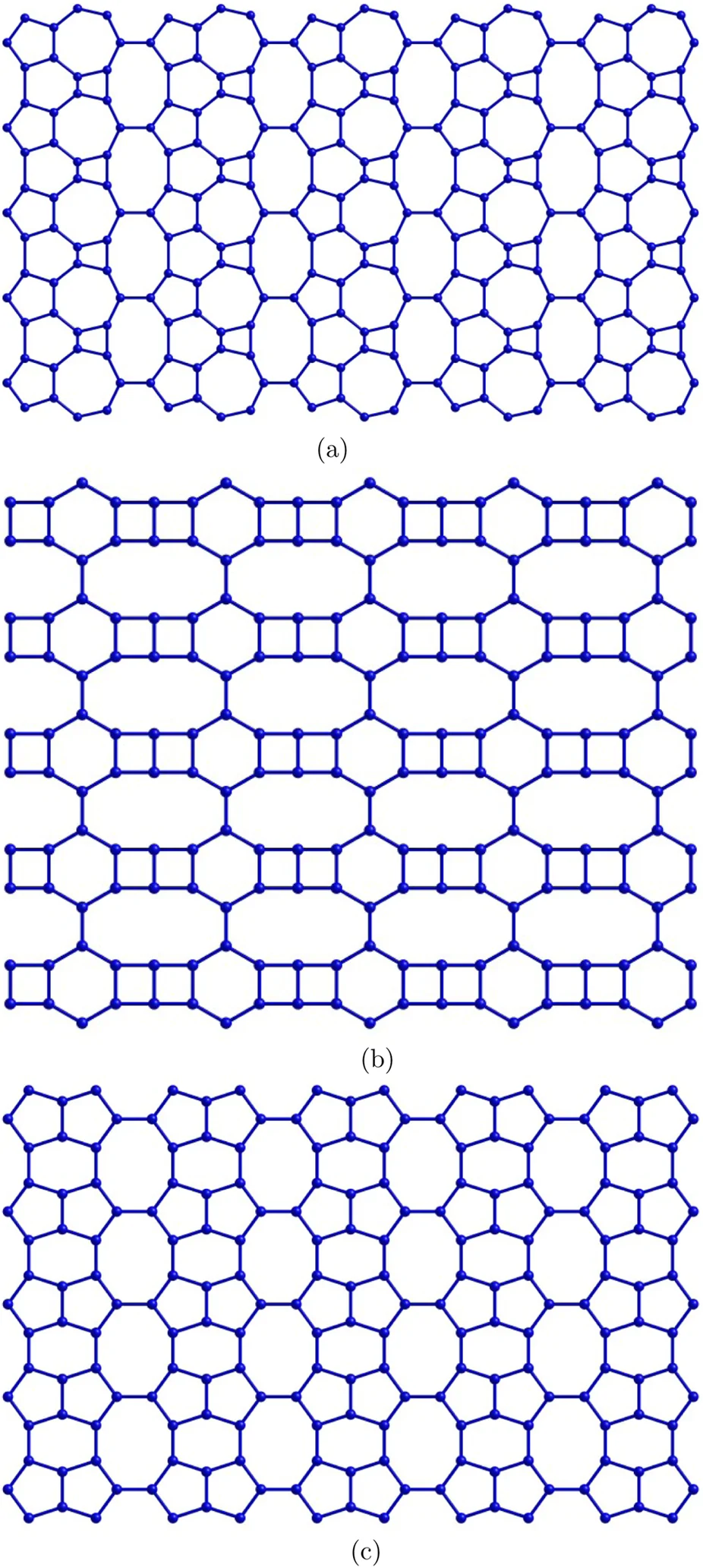
Molecular structure of graphene framework of dimension (5,5). **(a)** PHOTH-G(5, 5). **(b)** S-G(5, 5). **(c)** PH-G(5, 5).

The computed bond-additive modified reverse degree-based topological descriptors, required in identifying the edge partitions of graphene structures according to their normal degree classes, are illustrated in Table 2. Considering the complexity associated with each descriptor and its variable parameter, we explicitly illustrate the first Zagreb descriptor for PHOTH-G in Table 1 using a variable parameter k=2 of modified reverse. When the reversing parameter is fixed at k=2 , the classes of normal degree (2, 2), (2, 3), and (3, 3) are converted into (3, 3), (3, 2), and (2, 2) respectively.

$$\begin{array}{ll} M_{2}RM_{1}\left(\text{PHOTH}-\text{G}\left(m,n\right)\right) & =\left(2m+4\right)\left(3+3\right)+\left(4n+8m-8\right)\left(3+2\right)+\left(15mn-13m-5n+4\right)\left(2+2\right)\\ & =60mn \end{array}$$

**TABLE 2:** Vertex degree-based partition of edges in graphene.

Graphenes	(2, 2)	(2, 3)	(3, 3)
PHOTH-G (m,n)	2m+4	8m+4n−8	15mn−13m−5n+4
S-G (m,n)	2m+2	4m+4n−4	12mn−8m−5n+2
PH-G (m,n)	4	4m+8n−8	12mn−5m−10n+4

We computed the topological indices of the graphene framework utilizing the modified reverse degree topological descriptors presented in Table 1 and the vertex degree-based partition of edges in Table 2. The computed values are systematically presented in Tables 3–5 for reversing the parameters k=1,2and3 .

**TABLE 3:** PHOTH-G modified degree descriptors with reversing parameter k = 1,2,3.

TD	k=1	k=2	k=3
$M_{k}RM_{1}$	30mn+6m+2n	60mn	90mn−14n−42m
$M_{k}RM_{2}$	15mn+11m+3n+4	60mn+14m+4n+4	135mn−91m−33n+16
$M_{k}RF$	30mn+30m+10n	120mn+36m+12n	270mn−150m−50n
$M_{k}RGA$	15mn−3.458m−1.229n+0.458	15mn−3.164m−1.082n+0.162	15mn−4.072m−1.536n+1.0718
$M_{k}RBM$	45mn+17m+5n+4	120mn+14m+4n+4	225mn−133m−47n+16
$M_{k}RTM$	45mn+41m+13n+4	180mn+50m+16n+4	405mn−241m−83n+16
$M_{k}RHG$	15mn−8.728m−3.114n+1.229	3.75mn−1.721m−0.596n+0.138	1.666mn+2.864m+0.599n+2.135
$M_{k}RHTM$	5mn−3.488m−1.286n+0.738	0.625mn−0.348m−0.124n+0.047	0.185mn+0.813m+0.092n+1.075
$M_{k}RHBM$	5mn−3.142m−1.133n+0.516	0.937mn−0.477m−0.167n+0.047	0.333mn+0.949m+0.174n+0.851
$M_{k}RBMG$	45mn−2.715m−0.857n−0.284	60mn−6.071m−2.036n+0.071	75mn−26.668m−8.834n−0.331
$M_{k}RBMH$	45mn+53m+15n+16	240mn+102m+30n+24	675mn−169n−467m+80

**TABLE 4:** S-G modified degree descriptors with reversing parameter k = 1,2,3.

TD	k=1	k=2	k=3
$M_{k}RM_{1}$	24mn+4m+2n	48mn	72mn−28m−14n
$M_{k}RM_{2}$	12mn+8m+3n+2	48mn+10m+4n+2	108mn−58m−33n+8
$M_{k}RF$	24mn+20m+10n	96mn+24m+12n	216mn−100m−50n
$M_{k}RGA$	12mn−2.228m−1.228n+0.228	12mn−2.080m−1.080n+0.080	12mn−2.535m−1.535n+0.535
$M_{k}RBM$	36mn+12m+5n+2	96mn+10m+4n+2	180mn−86m−47n+8
$M_{k}RTM$	36mn+28m+13n+2	144mn+34m+16n+2	324mn−158m−83n+8
$M_{k}RHG$	12mn−6.979m−3.114n+1.979	3mn−1.124m−0.596n+0.069	1.333mn+2.265m+0.599n+1.067
$M_{k}RHTM$	4mn−2.202m−1.285n+0.369	0.5mn−0.224m−0.124n+0.023	0.148mn+0.721m+0.092n+0.537
$M_{k}RHBM$	4mn−2.008m−1.133n+0.258	0.75mn−0.310m−0.167n+0.024	0.266mn+0.774m+0.174n+0.425
$M_{k}RBMG$	36mn−1.8578m−0.8578n−0.14217	48mn−4.037m−2.037n+0.037	60mn−17.810m−8.810n−0.189
$M_{k}RBMH$	36mn+38m+15n+8	192mn+72m+30n+12	540mn−169n−298m+40

**TABLE 5:** PH-G modified degree descriptors with reversing parameter k = 1,2,3.

TD	k=1	k=2	k=3
$M_{k}RM_{1}$	24mn+2m+4n	48mn	72mn−14m−28n
$M_{k}RM_{2}$	12mn+3m+6n+4	48mn+4m+8n+4	108mn−33m−66n+16
$M_{k}RF$	24mn+10m+20n	96mn+12+24n	216mn−50m−100n
$M_{k}RGA$	12mn−1.228m−2.457n+0.457	12mn−1.080m−2.161n+0.161	12mn−1.536m−3.072n+1.072
$M_{k}RBM$	36mn+5m+10n+4	96mn+4m+8n+4	180mn−47m−94n+16
$M_{k}RTM$	36mn+13m+26n+4	144mn+16m+32n+4	324mn−83m−166n+16
$M_{k}RHG$	12mn−3.114m−6.228n+1.228	3mn−0.596m−1.193n+0.138	1.333mn+0.599m+1.19829n+2.135
$M_{k}RHTM$	4mn−1.285m−2.571n+0.738	0.5mn−0.124m−0.248n+0.047	0.148mn+0.092m+0.184n+1.075
$M_{k}RHBM$	4mn−1.133m−2.266n+0.516	0.75mn−0.167m−0.334n+0.048	0.267mn+0.175m+0.349n+0.851
$M_{k}RBMG$	36mn−0.8578m−1.7157n−0.2842	48mn−2.036m−4.072n+0.072	60mn−8.8342m−17.668n−0.3316
$M_{k}RBMH$	36mn+15m+30n+16	192mn+30m+60n+24	540mn−338n−169m+80

The calculated numerical values of the topological descriptors for the graphene structures are summarized in Tables 6–8 and shown in Figure 3. Among the frameworks, the PHOTH-G (m,n) structure generally has the highest descriptor values, reflecting its strong structural coherence. Notably, the modified reverse degree-based descriptors, including $MRM_{1}$ , $MRM_{2}$ , MRGA , MRF , MRHG , MRHTM , and MRBMG , consistently show lower values compared to other descriptor families across all graphene types. This suggests that, for large graphene frameworks, these descriptors may be preferred to reduce the complexity of mathematics occurred due to the rapid growth of further descriptor sets.

**TABLE 6:** Modified degree descriptors with reversing parameters k=1 .

TD	$M_{1}RM_{1}$	$M_{1}RM_{2}$	$M_{1}RF$	$M_{1}RGA$	$M_{1}RBM$	$M_{1}RTM$	$M_{1}RHG$	$M_{1}RHTM$	$M_{1}RHBM$	$M_{1}RBMG$	$M_{1}RBMH$
PHOTH-G(1,1)	38	33	70	10.7712	71	103	4.3856	0.96429	1.24167	41.14214	129
PHOTH-G(1,2)	70	51	110	24.5425	121	161	16.27124	4.67857	5.10833	85.28427	189
PHOTH-G(1,3)	102	69	150	38.3137	171	219	28.15685	8.39286	8.97500	129.426407	249
PHOTH-G(2,2)	136	92	200	51.0849	228	292	37.54247	11.19048	11.96667	172.56854	332
PHOTH-G(2,3)	198	125	270	79.8562	323	395	64.42809	19.90476	20.83333	261.71068	437
PHOTH-G(3,3)	294	181	390	121.3987	475	571	100.6993	31.41667	32.69167	393.99495	625
S-G(1,1)	30	25	54	8.771236	55	79	3.885618	0.880952	1.116667	33.142136	97
S-G(1,2)	56	40	88	19.54247	96	128	12.77124	3.595238	3.983333	68.284271	148
S-G(1,3)	82	55	122	30.31371	137	177	21.65685	6.309524	6.85	103.4264	199
S-G(2,2)	108	72	156	41.31371	180	228	31.15685	9.392857	9.975	138.4264	258
S-G(2,3)	158	99	214	64.08494	257	313	52.04247	16.10714	16.84167	209.5685	345
S-G(3,3)	234	143	306	97.85618	377	449	82.42809	25.90476	26.83333	315.7107	491
PH-G(1,1)	30	25	54	8.771236	55	79	3.885618	0.880952	1.116667	33.142136	97
PH-G(1,2)	58	43	98	18.31371	101	141	9.656854	2.309524	2.85	67.426407	163
PH-G(1,3)	86	61	142	27.85618	147	203	15.42809	3.738095	4.583333	101.7106	229
PH-G(2,2)	108	70	156	41.08494	178	226	30.54247	9.02381	9.716667	138.5685	250
PH-G(2,3)	160	100	224	62.62742	260	324	48.31371	14.45238	15.45	208.8528	352
PH-G(3,3)	234	139	306	97.39865	373	445	81.19933	25.16667	26.31667	315.9949	475

**TABLE 7:** Modified degree descriptors with reversing parameters k=2 .

TD	$M_{2}RM_{1}$	$M_{2}RM_{2}$	$M_{2}RF$	$M_{2}RGA$	$M_{2}RBM$	$M_{2}RTM$	$M_{2}RHG$	$M_{2}RHTM$	$M_{2}RHBM$	$M_{2}RBMG$	$M_{2}RBMH$
PHOTH-G(1,1)	60	82	168	10.9192	142	250	1.5699	0.19995	0.3413	51.9629	396
PHOTH-G(1,2)	120	146	300	24.8384	266	446	4.7231	0.7008	1.1117	109.9258	666
PHOTH-G(1,3)	180	210	432	38.7576	390	642	7.8763	1.2017	1.8822	167.8888	936
PHOTH-G(2,2)	240	280	576	51.6767	520	856	10.5017	1.6023	2.5096	223.8517	1248
PHOTH-G(2,3)	360	404	828	80.5959	764	1232	17.4049	2.7282	4.2176	341.8146	1758
PHOTH-G(3,3)	540	598	1224	122.4343	1138	1822	26.9335	4.2546	6.5529	515.7405	2580
S-G(1,1)	48	64	132	8.9192	112	196	1.3476	0.1753	0.2968	41.9629	306
S-G(1,2)	96	116	240	19.8384	212	356	3.7508	0.5511	0.8798	87.9258	528
S-G(1,3)	144	168	348	30.7576	312	516	6.1540	0.9270	1.4628	133.8888	750
S-G(2,2)	192	222	456	41.7576	414	678	8.6263	1.3267	2.0697	179.8888	984
S-G(2,3)	288	322	660	64.6767	610	982	14.0295	2.2026	3.4027	273.8517	1398
S-G(3,3)	432	476	972	98.5959	908	1448	21.9049	3.4782	5.3426	413.8146	2046
PH-G(1,1)	48	64	132	8.9192	112	196	1.3476	0.1753	0.2968	41.9629	306
PH-G(1,2)	96	120	252	18.7576	216	372	3.1540	0.4270	0.7128	85.8888	558
PH-G(1,3)	144	176	372	28.5959	320	548	4.9604	0.6788	1.1287	129.8146	810
PH-G(2,2)	192	220	456	41.6767	412	676	8.5572	1.3029	2.0457	179.8517	972
PH-G(2,3)	288	324	672	63.5151	612	996	13.3636	2.0546	3.2116	271.7775	1416
PH-G(3,3)	432	472	972	98.4343	904	1444	21.7668	3.4305	5.2946	413.7405	2022

**TABLE 8:** Modified degree descriptors with reversing parameters k=3 .

TD	$M_{3}RM_{1}$	$M_{3}RM_{2}$	$M_{3}RF$	$M_{3}RGA$	$M_{3}RBM$	$M_{3}RTM$	$M_{3}RHG$	$M_{3}RHTM$	$M_{3}RHBM$	$M_{3}RBMG$	$M_{3}RBMH$
PHOTH-G(1,1)	34	27	70	10.4641	61	97	7.2658	2.1662	2.3079	39.1658	119
PHOTH-G(1,2)	110	129	290	23.9282	239	419	9.5316	2.4435	2.8159	105.332	625
PHOTH-G(1,3)	186	231	510	37.3923	417	741	11.7974	2.7208	3.3238	171.497	1131
PHOTH-G(2,2)	248	308	680	49.8564	556	988	15.7299	3.6277	4.4317	228.663	1508
PHOTH-G(2,3)	414	545	1170	78.3205	959	1715	19.6624	4.0902	5.2730	369.829	2689
PHOTH-G(3,3)	642	859	1830	119.249	1501	2689	27.5273	5.4596	7.2222	568.161	4247
S-G(1,1)	30	25	66	8.4641	55	91	5.2658	1.4995	1.6413	33.1658	113
S-G(1,2)	88	100	232	18.9282	188	332	7.1983	1.7398	2.0825	84.3316	484
S-G(1,3)	146	175	398	29.3923	321	573	9.1308	1.9801	2.5238	135.4974	855
S-G(2,2)	204	258	564	40.3923	462	822	12.1308	2.7578	3.3905	186.4974	1266
S-G(2,3)	334	441	946	62.8564	775	1387	15.3966	3.1462	4.0984	297.6632	2177
S-G(3,3)	522	707	1494	96.3205	1229	2201	21.6624	4.3124	5.6730	459.8290	3499
PH-G(1,1)	30	25	66	8.464102	55	91	5.265812	1.499525	1.64127	33.16581	113
PH-G(1,2)	74	67	182	17.3923	141	249	7.797435	1.831909	2.257143	75.49742	315
PH-G(1,3)	118	109	298	26.32051	227	407	10.32906	2.164292	2.873016	117.829	517
PH-G(2,2)	204	250	564	39.85641	454	814	11.06325	2.220323	2.965079	186.6632	1226
PH-G(2,3)	320	400	896	60.78461	720	1296	14.9282	2.700855	3.847619	288.9948	1968
PH-G(3,3)	522	691	1494	95.24871	1213	2185	19.52735	3.237417	4.822222	460.1607	3419

**FIGURE 3:**
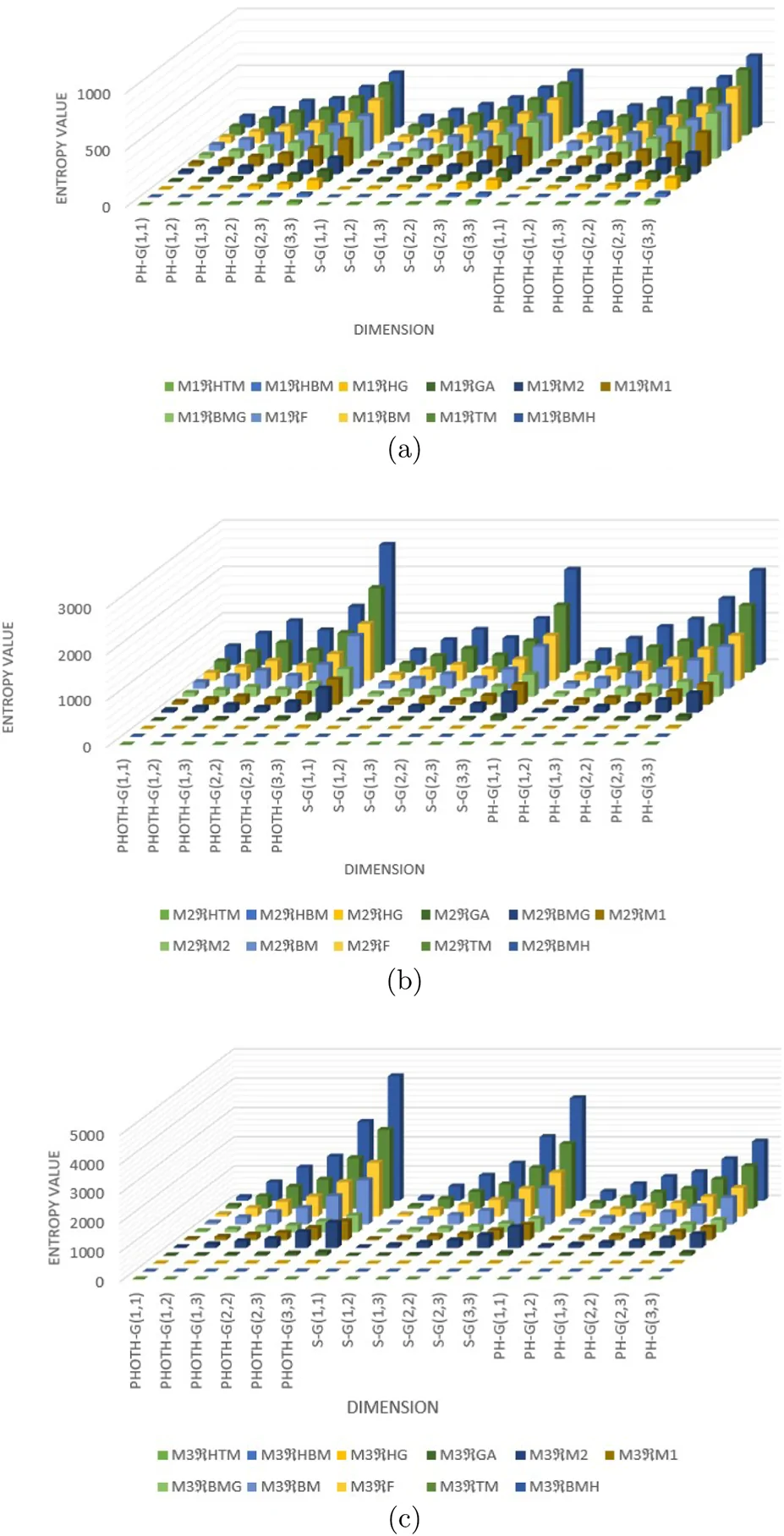
Graphical comparison of numerical descriptor values for all graphenes over varying *k* values. **(a)** *k* = 1. **(b)** *k* = 2. **(c)** *k* = 3.

A desirable topological descriptor should possess strong discriminating ability to distinguish between different structural formulas. One of the major challenges associated with topological descriptors (TDs) is that two or more distinct structures may yield identical descriptor values, thereby reducing their effectiveness in structural characterization. To evaluate the discriminating ability of the proposed descriptors, the sensitivity of the topological indices is employed, which is defined as follows:

$$S_{i}=\frac{N-N_{i}}{N},$$

where N denotes the total number of considered structures and $N_{i}$ represents the number of structures having the same value of the corresponding topological descriptor. A higher value of $S_{i}$ indicates greater discriminating power and improved robustness of the descriptor.

To assess the robustness and discriminating capability of the proposed modified reverse degree descriptors, a sensitivity analysis was performed for the considered graphene frameworks are summarized in Table 9.

**TABLE 9:** Sensitivity analysis.

Structure	Dim (m,n)	N	$N_{i}$	$S_{i}$
PHOTH-G (m,n)	(1,1)	70	2	0.9714
	(1,2)	110	2	0.9818
	(1,5)	338	2	0.9941
S-G (m,n)	(1,1)	30	2	0.9333
		25	2	0.9200
		55	2	0.9636
	(1,2)	88	2	0.9773
		96	2	0.9792
	(1,4)	250	2	0.9920
	(2,2)	258	2	0.9922
	(3,2)	328	2	0.9939
PH-G (m,n)	(1,1)	30	2	0.9333
		25	2	0.9200
		55	2	0.9636
	(1,2)	141	2	0.9858
	(2,1)	88	2	0.9773
		96	2	0.9792
	(2,2)	250	2	0.9920
	(2,3)	324	2	0.9938
	(4,1)	250	2	0.9920

## 4 Comparative analysis of entropy levels

The concept of entropy proposed by Shannon establishes a rigorous mathematical approach for characterizing uncertainty and randomness in systems characterized by probability distributions. For a discrete random variable $\left(x_{1},x_{2},\ldots ,x_{n}\right)$ with chances $\left(h\left(x_{1}\right),h\left(x_{2}\right),\ldots ,h\left(x_{n}\right)\right)$ , according to Shannon’s entropy (H) , it is expressed as:

$$H=-\overset{n}{\underset{i=1}{\sum }}h\left(x_{i}\right)\;\log_{2}h\left(x_{i}\right).$$

where $h\left(x_{i}\right)=\frac{N_{i}}{N}$ , $N_{i}$ be the number of occurrences of outcome $x_{i}$ , and N is the total number of outcomes (Rahul and Clement, 2023; Shannon, 1948). The information obtained from the system is measured by utilizing the base-2 logarithm to values of entropy expressed in bits. The above equation indicates similarity with thermodynamic entropy, which reflects the states of randomness in a physical system (Mowshowitz and Dehmer, 2012; Sabirov and O-sawa, 2012). Shannon’s entropy is occasionally relevant to conceptual systems, allowing the analysis of their complexity of structure through vertices and edges. Thermodynamic entropy is utilized in physics to assess the distribution of microstates within a system (Mowshowitz, 1968). Incorporating topological indices within an entropy framework appears to be an appropriate approach for determining molecular complexity. This approach employs topological descriptors (TDs) to mathematically describe molecular graph structures, particularly focusing on graph edges (Arockiaraj et al., 2023c). The probability associated with each edge ab∈E(G) of a molecular graph G , is defined by $\frac{f\left(ab\right)}{M_{k}RTD}$ , where $M_{k}RTD$ is the corresponding modified reverse degree-based descriptors, and f(ab) is a modified reverse degree-based function. The graph entropy is expressed as

$$I_{M_{k}RTD}=-\underset{ab\in E\left(G\right)}{\sum }\frac{f\left(ab\right)}{M_{k}RTD}\log_{2}\left(\frac{f\left(ab\right)}{M_{k}RTD}\right).$$

Further, the graph entropy equation can be simplified as follows:

$$I_{M_{k}RTD}=\log_{2}\left(M_{k}RTD\left(G\right)\right)-\frac{1}{M_{k}RTD\left(G\right)}\underset{ab\in E\left(G\right)}{\sum }f\left(ab\right)\log_{2}f\left(ab\right).$$

For the molecular graph of graphene, the simplified representation helps to compute the graph entropy using specific topological descriptors in the entropy formula as follows:

$$I_{M_{k}RM_{1}}=\log_{2}\left(M_{k}RM_{1}\right)-\frac{1}{M_{k}RM_{1}}\underset{ab\in E\left(G\right)}{\sum }f\left(ab\right)\;\log_{2}f\left(ab\right).$$

For example, the $M_{2}RM_{1}$ of the structure of PHOTH-G (m,n) is illustrated using the vertex degree–based partition of edges presented in Table 2, yields the entropy of PHOTH-G (m,n) for k=2 as follows:

$$\begin{array}{ll} I_{M_{2}RM_{1}}\left(\text{PHOTH}-\text{G}\left(m,n\right)\right) & =\log_{2}\left(60mn\right)-\frac{1}{60mn}\left[\left(2m+4\right)6\log_{2}\left(6\right)+\left(8m+4n-8\right)5\log_{2}\left(5\right)\right.\\ & \left.\;+\left(15mn-13m-5n+4\right)4\log_{2}\left(4\right)\right] \end{array}$$

For m,n=1 , we obtain:

$$I_{M_{2}RM_{1}}\left(\text{PHOTH}-\text{G}\left(1,1\right)\right)=\log_{2}\left(60\right)-\frac{1}{60}\left[147.49712\right]=3.4485$$

The entropy level of the graphene structures is determined using the methodology described above using the modified reverse degree indices. Recent literature compares degree indices for many chemical structures that include graphene, graphyne, and graphdiyne (Govardhan et al., 2024; Kavitha et al., 2021; Peter and Clement, 2024; Raja and Anuradha, 2024; Shanmukha et al., 2023). In this study, we focus on entropy measures based on modified reverse degree indices and examine their effectiveness and provide insights for future structural developments.

The calculated entropy values of the graphene framework when k=1,2 , and 3 are provided as Supplementary Tables S3–S11 in the supplementary information and the dynamic differences among graphenes are examined. Notably, the entropy values exhibit a consistent increase k=3 compared to k=1 and 2, with $M_{3}RBMG$ attaining the highest levels. However, direct comparisons of structural complexity are complicated by variations in the number of edges among the considered frameworks. We employ scaled bond-wise entropy (BIs) , where entropy is normalized by incorporating the number of edges in the graphene frameworks (Arockiaraj et al., 2023d). The bond-wise entropy BIs thus provides a more refined characterization of structural features and stability-related dynamics in graphene structures.

For instance, PHOTH-G(6, 1) has $I_{M_{2}RBMG}=6.9322$ , and |E(BMG(6,1))|=71 , then the bond-wise entropy is measured for PHOTH-G(6, 1) by the following formula.

$$BI_{M_{2}RBMG}\left(\text{BMG}\left(6,1\right)\right)=\frac{I_{M_{2}RBMG}\left(\text{BMG}\left(6,1\right)\right)}{\left|E\left(\text{BMG}\left(6,1\right)\right)\right|}=\frac{6.9322}{71}=0.09764$$

We now examine the bond range with suitably defined classes and evaluate the bond-wise entropy associated with the bi-Zagreb geometric index.

A comparative study of bond-wise entropies is reported in Table 10 and Figure 4. Since all graphene frameworks share identical bond ranges, bond-wise entropy enables a clear comparison of structural complexity on a per-bond basis. The S-G and PH-G frameworks show lower entropy values, indicating simpler structures, while PHOTH-G exhibits the highest normalized entropy. As the dimensions (m,n) increase, the normalized entropies of all frameworks gradually converge, suggesting similar complexity in large-scale graphene structures.

**TABLE 10:** Bond-wise entropy comparison k=2 .

(50–100)		(100–150)		(150–200)	
Dim (m,n)	BI	Dim (m,n)	BI	Dim (m,n)	BI
PHOTH-G(6,1)	0.097636	PHOTH-G(10,1)	0.064449	PHOTH-G(2,6)	0.047451
S-G(1,7)	0.091451	S-G(1,11)	0.063133	S-G(8,2)	0.046315
PH-G(7,1)	0.091451	PH-G(11,1)	0.063133	PH-G(2,8)	0.04629

(200–250)		(250–300)		(300–350)	
Dim (m,n)	BI	Dim (m,n)	BI	Dim (m,n)	BI
PHOTH-G(18,1)	0.039617	PHOTH-G(23,1)	0.032377	PHOTH-G(27,1)	0.028182
S-G(22,1)	0.038802	S-G(4,6)	0.031474	S-G(4,7)	0.02766
PH-G(1,22)	0.038757	PH-G(6,4)	0.03147	PH-G(7,4)	0.027657

(350–400)		(400–450)		(450–500)	
Dim (m,n)	BI	Dim (m,n)	BI	Dim (m,n)	BI
PHOTH-G(30,1)	0.025779	PHOTH-G(35,1)	0.022618	PHOTH-G(39,1)	0.020628
S-G(36,1)	0.025698	S-G(42,1)	0.022508	S-G(47,1)	0.020455
PH-G(1,36)	0.025698	PH-G(1,42)	0.022484	PH-G(1,47)	0.020433

**FIGURE 4:**
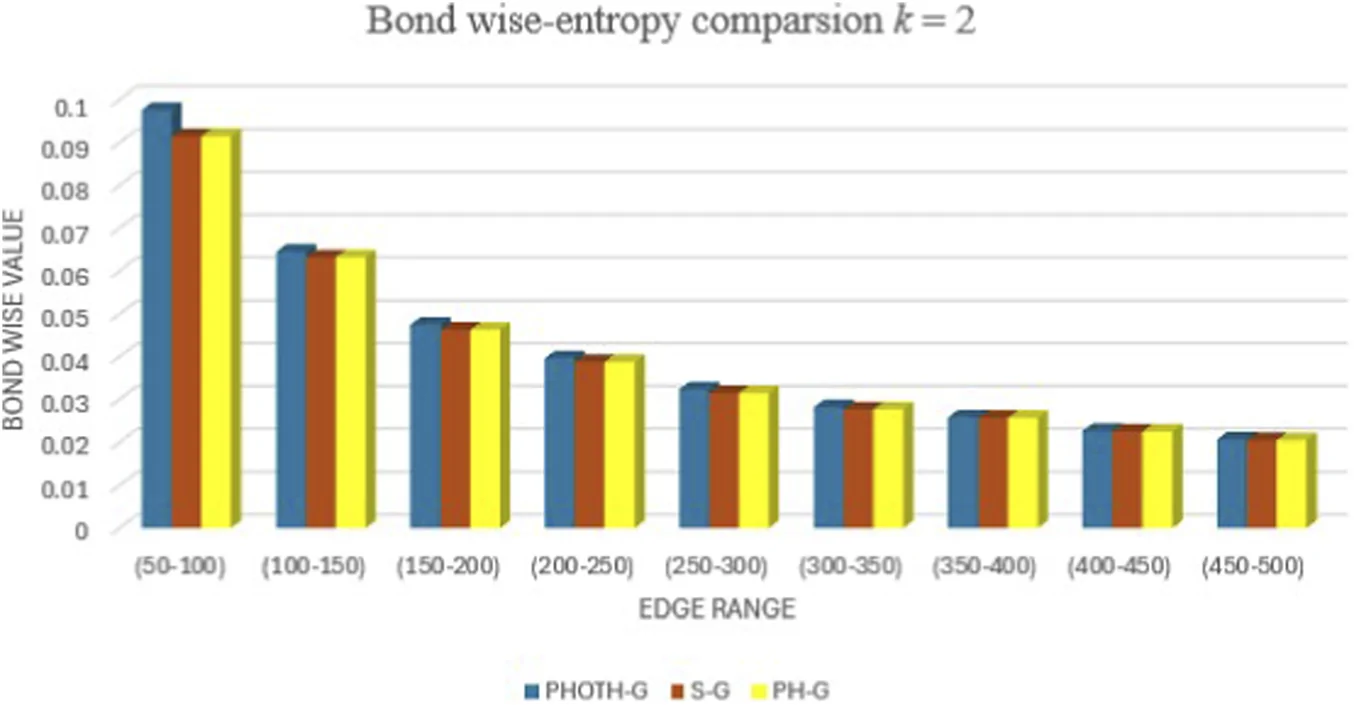
Graphical comparison of Bond-wise entropy comparison for all graphenes across a range of *k* = 2 values.

## 5 Estimating graph energy with regression models

Graph energy is a fundamental invariant that plays a crucial role in characterizing electronic properties and has a significant impact on chemical reactivity, materials science, energy applications, and optoelectronic systems. Furthermore, graph energy is associated with the total π -electronic energy. The adjacency matrices corresponding to the molecular structures are generated using the integrated software (Stevanović et al., 2021). Let the adjacency matrix of a graphene framework G of order |V(G)| be denoted by A(G) . The eigenvalues $\lambda_{1},\lambda_{2},\ldots ,\lambda_{m}$ , of A(G) comprise the spectrum of the graph G (Gutman, 1978). The graph energy $E_{\pi }\left(G\right)$ is then defined as the sum of the absolute eigenvalues $\left|\lambda_{i}\right|$ of the spectrum.

$$E_{\pi }\left(G\right)=\overset{\left|V\left(G\right)\right|}{\underset{i=1}{\sum }}|\lambda_{i}|$$

In the previous study, considerable research has concentrated on topological descriptors based on degree-based, degree-sum based, and distance-based as well as graph energies for a variety of molecular and crystalline structures (Balasubramanian, 2023; Govardhan and Roy, 2023; Hayat et al., 2020; Hayat et al., 2021a; Hayat et al., 2021b; Taherpour and Mohammadinasab, 2010). However, this study investigates the correlation between the graph energy $E_{\pi }\left(G\right)$ of the graphene frameworks and a set of modified reverse degree descriptors. The observed high correlation coefficient between these $E_{\pi }\left(G\right)$ and descriptors highlights the significance of graph energy in Hückel molecular orbital theory (Gutman et al., 2017).

Computing the graph energy for higher-order dimensions (m,n) using the software newGRAPH can be complicated, as it involves constructing the adjacency matrix for higher-order dimensions. Despite this, we construct regression models that reliably predict graph energy for higher-order dimension cases by combining all three distinct graphene frameworks into a dataset. The energy values for fixed (m,n) graphenes obtained from the software newGRAPH are presented in Table 11. Tables 6–8 illustrates the linear regression equations used in predicting the graph energy from the topological descriptor values computed for each framework. Furthermore, linear regression equations and quadratic regression equations are compared to evaluate their relative predictive accuracy.

**TABLE 11:** Energy values for graphenes obtained from newGRAPH.

$E_{\pi }\left(m,n\right)$	PHOTH-G	S-G	PH-G	$E_{\pi }\left(m,n\right)$	PHOTH-G	S-G	PH-G
(1,1)	13.36352	10.38118	10.45564	(3,3)	129.5807	101.6945	105.3142
(1,2)	27.66895	21.68099	21.28353	(3,4)	174.1554	136.5518	140.9136
(1,3)	42.18363	32.95785	32.11307	(4,1)	54.73894	42.78641	46.00211
(1,4)	56.77328	44.2497	42.94267	(4,2)	113.5335	89.40235	93.73893
(2,1)	27.15259	21.18108	22.54193	(4,3)	173.2441	136.0848	141.5097
(2,2)	56.29279	44.24582	45.88564	(4,4)	232.8377	182.7454	189.2867
(2,3)	85.8704	67.31266	69.23396	(5,1)	68.53231	53.58911	57.64382
(2,4)	115.4404	90.37528	92.63694	(5,2)	142.1546	111.9831	117.6739
(3,1)	40.94456	31.98372	34.10933	(5,3)	216.9611	170.4797	177.7682
(3,2)	84.91197	66.82272	69.71579	(5,4)	291.5309	228.9464	237.8192

### 5.1 Analysis of spectral properties in graphene framework

In this section, spectral properties of the graphene framework are analyzed by graph spectral descriptors. The spectral and energetic descriptors, including the ${\left(E_{\pi }\right)}_{\text{perbond}}$ , delocalization energy, and spectral diameter are derived from the combinatorial analysis of graph spectra. These descriptors provide valuable insights into the electronic, thermodynamic, and kinetic stability of the graphene frameworks under investigation. Among them, the delocalization energy measures the additional stabilization arising from the delocalization of π -electrons throughout the conjugated network. The delocalization energy per bond is defined as $E_{\left(Deloc\right)}=E_{\pi }-|V\left(G\right)|$ . The spectral diameter (SD) provides a measure of the spread of the eigenvalues of a graph and is defined as $SD=\lambda_{\max}-\lambda_{\min}$ , where $\lambda_{\max}$ and $\lambda_{\min}$ denote the largest and smallest eigenvalues of the adjacency matrix, respectively. All spectral descriptors were computed using newGRAPH and MATLAB (MATLAB version, 2022; Stevanović et al., 2021). The spectral descriptors ${\left(E_{\pi }\right)}_{\text{perbond}}$ , ${\left(E_{\text{Deloc}}\right)}_{\text{perbond}}$ , and spectral diameter (SD) for the PHOTH-G, S-G, and PH-G graphene frameworks at the dimensions (m,n) are given in Table 12. Both ${\left(E_{\pi }\right)}_{\text{perbond}}$ and ${\left(E_{\text{Deloc}}\right)}_{\text{perbond}}$ increase with increasing graph dimensions, indicating enhanced π -electron delocalization and thermodynamic stability for larger graphene frameworks. The gradual increase in these energetic descriptors suggests that larger graphene frameworks are more resonance stabilized.

**TABLE 12:** Spectral Properties of graphene frameworks.

Dim (m,n)	PHOTH-G			S-G			PH-G		
​	${\left(E_{\pi }\right)}_{\text{perbond}}$	${\left(E_{\text{Deloc}}\right)}_{\text{perbond}}$	SD	${\left(E_{\pi }\right)}_{\text{perbond}}$	${\left(E_{\text{Deloc}}\right)}_{\text{perbond}}$	SD	${\left(E_{\pi }\right)}_{\text{perbond}}$	${\left(E_{\text{Deloc}}\right)}_{\text{perbond}}$	SD
(1,1)	3.36352	0.336352	4.4056	2.38118	0.297648	4.7113	2.45564	0.306955	4.3429
(1,2)	7.66895	0.383448	5.2953	5.68099	0.355062	5.393	5.28353	0.330221	4.6376
(1,3)	12.18363	0.406121	5.3859	8.95785	0.373244	5.5159	8.11307	0.338045	4.7205
(1,4)	16.77328	0.419332	5.4234	12.2497	0.382803	5.5624	10.94267	0.341958	4.7525
(1,5)	21.28939	0.425788	5.4527	15.53694	0.388424	5.5974	13.77227	0.344307	4.7768
(2,1)	7.15259	0.35763	4.6092	5.18108	0.323818	5.0248	6.54193	0.408871	5.0515
(2,2)	16.29279	0.40732	5.446	12.24582	0.382682	5.5974	13.88564	0.433926	5.2872
(2,3)	25.8704	0.431173	5.5671	19.31266	0.402347	5.7226	21.23396	0.442374	5.3581
(2,4)	35.44043	0.443005	5.6181	26.37528	0.412114	5.7703	28.63694	0.447452	5.3864
(2,5)	45.02029	0.450203	5.6587	33.45966	0.418246	5.8064	36.03903	0.450488	5.4082
(3,1)	10.94456	0.364819	4.664	7.98372	0.332655	5.129	10.10933	0.421222	5.1387
(3,2)	24.91197	0.4152	5.4918	18.82272	0.39214	5.6744	21.71579	0.452412	5.4197
(3,3)	39.58066	0.439785	5.6174	29.69446	0.412423	5.7994	33.3142	0.462697	5.4948
(3,4)	54.15535	0.451295	5.6694	40.55179	0.422414	5.8473	44.91363	0.46785	5.5245
(3,5)	68.75367	0.458358	5.7105	51.44106	0.428676	5.8837	56.51359	0.470947	5.5473
(4,1)	14.73894	0.368474	4.685	10.78641	0.337075	5.172	14.00211	0.437566	5.1743
(4,2)	33.53349	0.419169	5.51	25.40235	0.396912	5.7075	29.73893	0.464671	5.4747
(4,3)	53.24406	0.443701	5.6371	40.0848	0.41755	5.8325	45.50967	0.474059	5.551
(4,4)	72.83768	0.455236	5.6895	54.74537	0.427698	5.8325	61.28673	0.478803	5.581
(4,5)	92.4876	0.462438	5.7308	69.43932	0.433996	5.9169	77.08649	0.481791	5.6041
(5,1)	18.53231	0.370646	4.695	13.58911	0.339728	5.1935	17.64382	0.441096	5.1922
(5,2)	42.15457	0.421546	5.5189	31.98307	0.399788	5.7245	37.6739	0.470924	5.5024
(5,3)	66.96106	0.446407	5.6468	50.47968	0.420664	5.8495	57.7682	0.481402	5.5792
(5,4)	91.53085	0.457654	5.6993	68.9464	0.430915	5.8975	77.81921	0.48637	5.6094
(5,5)	116.20466	0.464819	5.7407	87.44381	0.437219	5.934	97.90164	0.489508	5.6326

Among the graphene frameworks considered, Both (*E_π_*)_per_ bond and (*E_Deloc_*)_per bond_ increase with increasing graph dimensions for each graphene framework. The gradual increase in these energetic descriptors suggests that larger graphene frameworks exhibit greater resonance stabilization. In addition, the spectral diameter (SD) increases progressively with the graph dimensions for all three frameworks, suggesting an increasing spread of the eigenvalue spectrum as the structures become larger. These observations demonstrate the strong dependence of the spectral and energetic features of graphene frameworks on the size of the graph and its structural topology.

### 5.2 Regression models for prediction of spectral properties

In this section, we systematically analyze the relationships between topological descriptors and spectral characteristics of various graphene frameworks.

The equations used to formulate the predictive equation models are presented below.

Linear equation:Y=sX+p

Here, X and Y represent the corresponding topological descriptor and the spectral property to be predicted, respectively. The parameters s correspond to the slope coefficient and p denote the constant coefficient of the regression line. The standard error (S.E) is expected to be minimal and should have a sufficiently high F-value. The correlation coefficient (r) measures the strength of the linear relationship and also the overall quality of fit. The correlation measures are analyzed and discussed, including statistical parameters such as $r^{2}$ and adjusted $r^{2}$ . Further, Mean Absolute Error (MAE), Mean Squared Error (MSE), Root Mean Squared Error (RMSE), and Mean Absolute Percentage Error (MAPE) are computed to evaluate the regression equations, where lower values mean better agreement between observed and predicted values.

We conduct the regression analysis to determine the most effective topological descriptor in the graphene framework. This analysis examines the relationship among the graph energy values presented in Table 11. The molecular descriptors are listed in Tables 6–8, with particular emphasis on descriptors that exhibit strong positive correlations. To enhance predictive accuracy, each graphene framework is analyzed individually. The regression analysis for the PHOTH-G *(m, n)*, S-G *(m, n)*, and PH-G *(m, n)* frameworks for *k* = 1, 2 and 3 are provided as Supplementary Tables S12–S20 in the supplementary information.

When considering parameters for effective prediction modeling, the BMG index $\left(M_{1}RBMG\right)$ demonstrated the highest F value and the lowest standard error (S.E) , indicating its suitability as the most effective predictive descriptor. The low values of MAE, MSE, RMSE, and MAPE indicate that all regression equations predict the energy values with high accuracy. Furthermore, Table 13 presents a comparative assessment of the predictive ability for BMG descriptor-based linear regression models for the normal degree (ND) and the modified degree reversed parameters k=1 , 2 and 3 with the corresponding scatter plots representation is shown in Figure 5.

**TABLE 13:** Comparison regression equation of graphenes.

Structure		r	adj $\left(r^{2}\right)$	S.E	F	MAE	MSE	RMSE	MAPE (%)	Equation
PHOTH-G (m,n)	ND	0.999	0.999	2.612	31785.491	2.0123	6.2846	2.5069	3.6360	0.208(BMG)+6.871
	k=1	1.000	1.000	0.331	1976067.07	0.2663	0.1138	0.3373	0.4485	$0.331\left(M_{1}RBMG\right)-0.845$
	k=2	1.000	1.000	0.327	2030815.8	0.4007	0.1843	0.4293	0.5046	$0.251\left(M_{2}RBMG\right)+0.452$
	k=3	0.999	0.999	3.639	16355.639	2.9056	12.2943	3.5063	5.4046	$0.211\left(M_{3}RBMG\right)+10.305$
S-G (m,n)	ND	1.000	1.000	1.337	74840.432	0.9577	1.7076	1.3067	2.4954	0.203(BMG)+4.717
	k=1	1.000	1.000	0.271	1827006.06	0.1902	0.06475	0.2596	0.5193	$0.325\left(M_{1}RBMG\right)-0.897$
	k=2	0.983	0.982	10.029	1307.414	3.5814	92.6008	9.6229	5.3354	$0.244\left(M_{2}RBMG\right)+2.492$
	k=3	0.999	0.999	1.942	35466.165	1.4032	3.5185	1.8785	3.6466	$0.206\left(M_{3}RBMG\right)+6.81$
PH-G (m,n)	ND	1.000	1.000	1.147	109566.471	0.8042	1.2362	1.1118	2.0976	0.211(BMG)+4.478
	k=1	1.000	1.000	0.716	281643.023	0.5061	0.4829	0.6949	1.1835	$0.337\left(M_{1}RBMG\right)-1.382$
	k=2	1.000	1.000	0.455	697499.183	0.3188	0.9138	0.4403	0.6889	$0.255\left(M_{2}RBMG\right)-0.356$
	k=3	1.000	1.000	1.713	49130.303	1.2205	2.7197	1.6491	3.1793	$0.214\left(M_{3}RBMG\right)+6.613$

**FIGURE 5:**
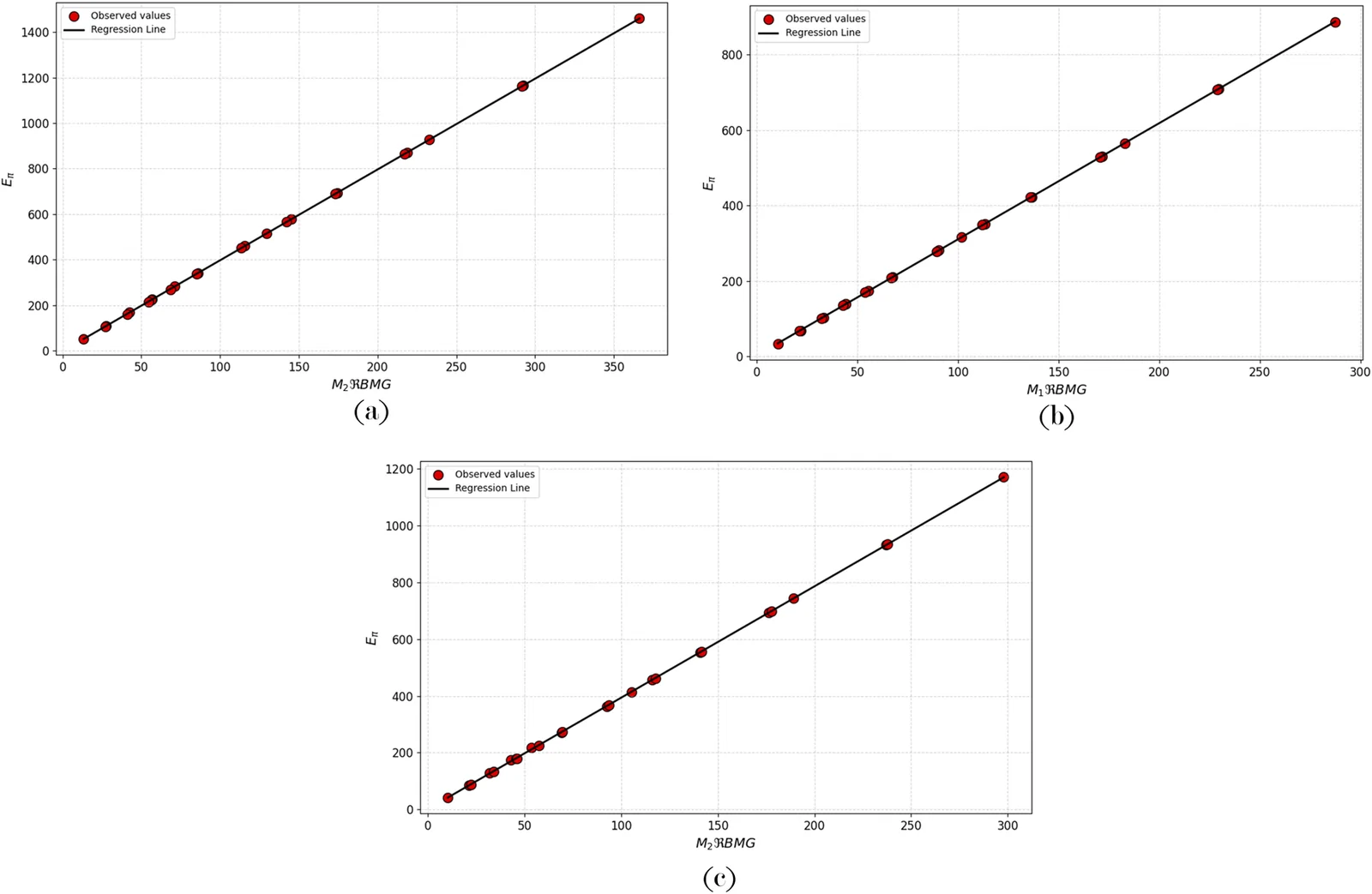
Scatter plots and regression lines for graph energy prediction. **(a)** PHOTH-G, **(b)** S-G, **(c)** PH-G.

The regression equation k=1 provides more accurate predictions of energy values compared to other k values, as shown in Table 14. Moreover, the BMG descriptor $M_{1}RBMG$ consistently emerges as the most effective predictor of the graph energies across all other graphene frameworks.

**TABLE 14:** Comparison graph energy of graphenes.

Structure		$E_{\pi }$	ND	k=1	k=2	k=3
PHOTH-G (m,n)	(1,5)	71.28939	73.1845	71.21723	71.68947	74.41293
	(2,5)	145.02029	146.8010	144.7933	145.4649	147.9109
	(3,5)	218.75367	220.4176	218.3694	219.2402	221.4089
	(4,5)	292.4876	294.0342	291.9455	293.0156	294.9068
	(5,5)	366.20466	367.6507	365.5216	366.791	368.4048
S-G (m,n)	(1,5)	55.53694	55.63237	55.55897	57.59077	55.80278
	(2,5)	113.45966	113.6828	113.4552	115.1657	113.9289
	(3,5)	171.44106	171.7333	171.3514	172.7407	172.0551
	(4,5)	229.43932	229.7838	229.2476	230.3156	230.1813
	(5,5)	287.44381	287.8343	287.1437	287.8906	288.3074
PH-G (m,n)	(1,5)	53.77227	51.46059	56.0021	55.14891	49.94635
	(2,5)	116.03903	113.2758	116.373	115.8295	112.2558
	(3,5)	176.51359	175.0909	176.7439	176.51	174.5653
	(4,5)	237.08649	236.9061	237.1148	237.1905	236.8748
	(5,5)	297.90164	298.7213	297.4857	297.8711	299.1843

Table 15 presents a comparison of the actual and predicted graph energy values for higher-dimensional graphene frameworks.

**TABLE 15:** Comparison graph energy of higher-dimensional.

Structure		$E_{\pi }$	ND	k=1	k=2	k=3
PHOTH-G (m,n)	(1,6)	85.78052	87.3207	85.8283	86.2382	88.3739
	(2,6)	174.62263	176.5373	174.2994	175.0736	177.6969
	(3,6)	263.36772	265.7540	262.7705	263.9091	267.0203
	(4,6)	352.10241	354.9715	351.2430	352.7456	356.3429
	(5,6)	440.85276	444.1868	439.7127	364.2715	445.6655
S-G (m,n)	(1,6)	66.82858	66.3838	66.9802	68.8057	66.3429
	(2,6)	136.52892	136.6143	145.2687	138.0927	136.8291
	(3,6)	206.31146	206.8448	206.1726	207.3796	207.3153
	(4,6)	276.11514	277.0760	275.7687	276.6675	277.8009
	(5,6)	345.92572	347.3059	345.3645	345.9537	348.2880
PH-G (m,n)	(1,6)	64.60187	61.1510	67.5559	66.3500	59.0053
	(2,6)	139.42134	135.6261	140.0588	139.2706	134.1548
	(3,6)	212.1342	210.1013	212.5617	212.1911	209.3043
	(4,6)	284.89744	284.5763	285.0646	285.1114	673.1674
	(5,6)	357.98499	359.0508	357.5668	358.0312	359.6039

Table 15 and Figure 6 demonstrate that the energy values predicted by the regression model are closely aligned with actual graph energies computed using the newGRAPH software. This capability enables accurate prediction of the graph energy of a higher-dimensional distinct graphene.

**FIGURE 6:**
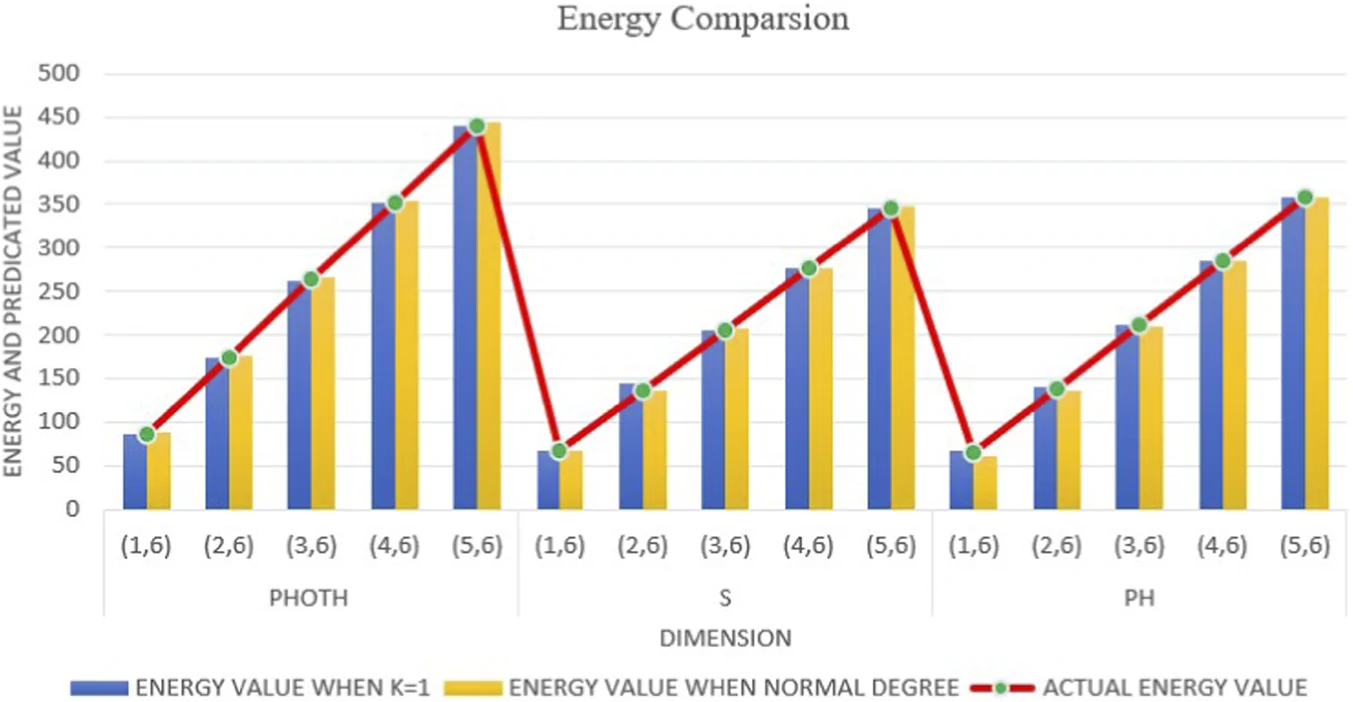
Energy comparison.

## 6 Cross-validation of QSPR linear regression models

The robustness and predictive performance of the developed models were evaluated using the calculated correlation coefficient $\left(Q_{\text{LOO}}^{2}\right)$ and Root Mean Square Error of Cross Validation (RMSECV). These models are widely used for internal validation parameters in QSPR/QSAR studies. In this procedure, each data point is systematically excluded, and the model is reconstructed using the remaining observations to assess the prediction ability of the omitted sample. The high $\left(Q_{\text{LOO}}^{2}\right)$ values and low RMSECV values indicates strong model fit and predictive power across most properties. The $Q_{\text{LOO}}^{2}$ value is calculated as:

$$Q_{\text{LOO}}^{2}=1-\frac{\sum_{i=1}^{m}{\left(y_{i}-{\hat{y}}_{i,LOO}\right)}^{2}}{\sum_{i=1}^{m}{\left(y_{i}-\bar{y}\right)}^{2}}$$

where $y_{i}$ represents the actual response value, ${\hat{y}}_{i,LOO}$ denotes the cross-validated predicted value of the $i^{th}$ observation, $\bar{y}$ is the mean response value, and m represents the total number of structures. The RMSECV value is expressed as:

$$RMSECV=\sqrt{\frac{1}{m}\overset{m}{\underset{i=1}{\sum }}{\left(y_{i,obs}-y_{i,pred\left(CV\right)}\right)}^{2}}$$

where $y_{i,obs}$ and $y_{i,pred\left(CV\right)}$ represent the observed and cross-validated predicted values, respectively.

The results presented in Table 16 show high $Q_{\text{LOO}}^{2}$ values and relatively low RMSECV values, indicating the reliability and strong predictive capability of the developed models. The $Q_{\text{LOO}}^{2}$ and RMSECV values obtained from leave-one-out cross-validation confirm the accuracy and stability of the regression models.

**TABLE 16:** $Q_{\mathrm{LOO}}^{2}$ and RMSECV values of graphene.

Structure	Descriptor	$Q_{LOO}^{2}$	RMSECV
PHOTH-G (m,n)	ND	0.999165	2.691878
	k=1	0.999986	0.346695
	k=2	0.999987	0.335146
	k=3	0.998338	3.797717
S-G (m,n)	ND	0.999640	1.388737
	k=1	0.999985	0.284574
	k=2	0.981140	10.047693
	k=3	0.999240	2.016453
PH-G (m,n)	ND	0.999749	1.202825
	k=1	0.999906	0.735275
	k=2	0.999962	0.466666
	k=3	0.999445	1.790516

## 7 Conclusion

An analysis was performed by computing modified reverse degree-based topological descriptors for certain graphene frameworks. The entropy levels of each graphene were also assessed and contrasted with those obtained using the bond-wise scaled entropy approach. An efficient linear regression model was built to predict the graph energy of different structural frameworks in order to reduce computational complexity and guarantee polynomial time performance. The robustness and predictive capability of the developed models were further validated using leave-one-out cross-validation (LOOCV). The combined application of graph-theoretical and statistical methodologies described here offers significant potential for advancing machine learning applications in computational chemistry and enhancing QSAR/QSPR modeling in material design and development.

## Data Availability

The original contributions presented in the study are included in the article/Supplementary Material, further inquiries can be directed to the corresponding author.
