# Supplementary material for: Comparison of degree and modified reverse degree descriptors incorporating entropy and graph energy analysis for graphene allotropes
Source: Front Chem. 2026 Oct 22;14:1835238. doi: 10.3389/fchem.2026.1835238 (PMC13737588; doi:10.3389/fchem.2026.1835238)
Supplement: Supplementary file 1 [file Supplementaryfile1.pdf]

# Comparison of Degree and Modified Reverse Degree Descriptors Incorporating Entropy and Graph Energy Analysis for Graphene Allotropes

**M. Regina Celine**[a], **A. Berin Greeni**[a,*]

[a]School of Advanced Sciences, Vellore Institute of Technology, Chennai 600127, India

Supplementary Information

*Corresponding author: beringreeni.a@vit.ac.in

**Table S1:** List of Symbols and Abbreviations

| **Symbol** | **Description** |
|---|---|
| $A(G)$ | Adjacency matrix of graph $G$. |
| adj. $r^2$ | Adjusted coefficient of determination. |
| $BIs$ | Scaled bond-wise entropy. |
| $d_a$ | Degree of vertex $a$. |
| $E(G)$ | Edge set of graph $G$. |
| $E_{\text{Deloc}}$ | Delocalization energy. |
| $E_\pi(G)$ | Graph energy. |
| $G$ | Graph. |
| $H(G)$ | Shannon entropy of graph $G$. |
| $IM_k\mathfrak{R}TI(G)$ | Entropy of a modified reverse topological index. |
| $k$ | Parameter of the modified reverse degree descriptor. |
| $\|E(G)\|$ | Number of edges (size of $G$). |
| $\|V(G)\|$ | Number of vertices (order of $G$). |
| $\lambda_i$ | $i^{\text{th}}$ eigenvalue of the adjacency matrix $A(G)$. |
| MAE | Mean Absolute Error. |
| MAPE | Mean Absolute Percentage Error. |
| $M_k\mathfrak{R}d_a$ | Modified reverse degree of vertex $a$. |
| $M_k\mathfrak{R}BM$ | Modified Bi-Zagreb index. |
| $M_k\mathfrak{R}BMG$ | Modified Bi-Zagreb–Geometric index. |
| $M_k\mathfrak{R}BMH$ | Modified Bi-Zagreb–Harmonic index. |
| $M_k\mathfrak{R}F$ | Modified Forgotten index. |
| $M_k\mathfrak{R}GA$ | Modified Geometric–Arithmetic index. |

**Table S2:** List of Symbols and Abbreviations

| Symbol | Description |
|---|---|
| $M_k\mathfrak{R}HBM$ | Modified Harmonic–Bi-Zagreb index. |
| $M_k\mathfrak{R}HG$ | Modified Harmonic–Geometric index. |
| $M_k\mathfrak{R}HTM$ | Modified Harmonic–Tri-Zagreb index. |
| $M_k\mathfrak{R}M_1$ | Modified First Zagreb index. |
| $M_k\mathfrak{R}M_2$ | Modified Second Zagreb index. |
| $M_k\mathfrak{R}TM$ | Modified Tri-Zagreb index. |
| MSE | Mean Squared Error. |
| ND | Normal degree-based topological descriptors. |
| $p$ | Constant (intercept) coefficient of the regression line. |
| $Q^2_{\text{LOO}}$ | Leave-one-out cross-validation coefficient. |
| QSPR | Quantitative Structure–Property Relationship. |
| $r$ | Correlation coefficient. |
| RE | Resonance energy. |
| RMSE | Root Mean Squared Error. |
| RMSECV | Root Mean Square Error of Cross-Validation. |
| $s$ | Slope coefficient of the regression line. |
| SD | Spectral diameter. |
| S.E | Standard error. |
| $\Delta(G)$ | Maximum degree of $G$. |
| $\bar{y}$ | Mean of the observed response values. |
| $\hat{y}_{i,\text{LOO}}$ | Cross-validated predicted value of the $i^{\text{th}}$ observation. |
| $y_i$ | Observed response value. |

**Table S3:** Entropy Values for PHOTH-G$(m, n)$ at $k = 1$

| $k=1$ | $I_{M_1\Re M_1}$ | $I_{M_1\Re M_2}$ | $I_{M_1\Re F}$ | $I_{M_1\Re GA}$ | $I_{M_1\Re BM}$ | $I_{M_1\Re TM}$ | $I_{M_1\Re HG}$ | $I_{M_1\Re HTM}$ | $I_{M_1\Re HBM}$ | $I_{M_1\Re BMG}$ | $I_{M_1\Re BMH}$ |
|---|---|---|---|---|---|---|---|---|---|---|---|
| $(1,1)$ | 3.431624 | 3.347424 | 3.380161 | 3.458859 | 3.400544 | 3.371179 | 3.283319 | 3.02429 | 3.194422 | 3.80759 | 3.321602 |
| $(1,2)$ | 4.585867 | 4.417523 | 4.427931 | 4.64332 | 4.528939 | 4.426364 | 4.460092 | 4.265663 | 4.391132 | 4.834288 | 4.330934 |
| $(1,3)$ | 5.230674 | 5.065046 | 5.060047 | 5.284877 | 5.177102 | 5.063427 | 5.139935 | 4.989594 | 5.087445 | 5.428132 | 4.966135 |
| $(1,4)$ | 5.677446 | 5.523403 | 5.507991 | 5.727402 | 5.628827 | 5.514672 | 5.602745 | 5.47217 | 5.557853 | 5.847606 | 5.422827 |
| $(1,5)$ | 6.019029 | 5.87615 | 5.853347 | 6.065574 | 5.97461 | 5.862353 | 5.953202 | 5.833764 | 5.912642 | 6.172195 | 5.776843 |
| $(2,1)$ | 4.492445 | 4.391118 | 4.427632 | 4.522937 | 4.456934 | 4.418121 | 4.364973 | 4.131095 | 4.289038 | 4.801128 | 4.35569 |
| $(2,2)$ | 5.645711 | 5.480084 | 5.475085 | 5.699915 | 5.59214 | 5.478465 | 5.554972 | 5.404632 | 5.502483 | 5.84317 | 5.381173 |
| $(2,3)$ | 6.290479 | 6.133784 | 6.113139 | 6.339394 | 6.241886 | 6.121417 | 6.230471 | 6.123196 | 6.192952 | 6.441885 | 6.025397 |
| $(2,4)$ | 6.737301 | 6.59492 | 6.566357 | 6.780942 | 6.694171 | 6.577124 | 6.690991 | 6.603156 | 6.660441 | 6.86375 | 6.487829 |
| $(2,5)$ | 7.078939 | 6.949157 | 6.915895 | 7.118548 | 7.040196 | 6.928141 | 7.040077 | 6.963237 | 7.01352 | 7.18976 | 6.845813 |
| $(3,1)$ | 5.098653 | 4.997626 | 5.031999 | 5.128667 | 5.063909 | 5.023289 | 4.980984 | 4.757309 | 4.910187 | 5.384036 | 4.960271 |
| $(3,2)$ | 6.25169 | 6.092876 | 6.08118 | 6.30326 | 6.201148 | 6.086686 | 6.171804 | 6.03481 | 6.124431 | 6.43109 | 5.992394 |
| $(3,3)$ | 6.896486 | 6.748465 | 6.722536 | 6.942084 | 6.851288 | 6.732474 | 6.845776 | 6.751618 | 6.812946 | 7.031403 | 6.640332 |
| $(3,4)$ | 7.343347 | 7.210383 | 7.178457 | 7.383326 | 7.303679 | 7.19026 | 7.30553 | 7.230753 | 7.279493 | 7.454054 | 7.10513 |
| $(3,5)$ | 7.685018 | 7.565006 | 7.530085 | 7.720754 | 7.649732 | 7.542812 | 7.654164 | 7.590363 | 7.632026 | 7.780532 | 7.464751 |
| $(4,1)$ | 5.524504 | 5.425046 | 5.457505 | 5.553982 | 5.490628 | 5.449427 | 5.411932 | 5.193415 | 5.343732 | 5.798064 | 5.387088 |
| $(4,2)$ | 6.677446 | 6.523403 | 6.507991 | 6.727402 | 6.628827 | 6.514672 | 6.602745 | 6.47217 | 6.557853 | 6.847606 | 6.422827 |
| $(4,3)$ | 7.322264 | 7.179882 | 7.15132 | 7.365905 | 7.279134 | 7.162086 | 7.275953 | 7.188118 | 7.245403 | 7.448713 | 7.072791 |
| $(4,4)$ | 7.769149 | 7.642152 | 7.608826 | 7.806997 | 7.73156 | 7.621055 | 7.735327 | 7.666852 | 7.711488 | 7.871755 | 7.538879 |
| $(4,5)$ | 8.110839 | 7.996939 | 7.96167 | 8.144339 | 8.077615 | 7.974476 | 8.083738 | 8.026234 | 8.063755 | 8.198465 | 7.899392 |
| $(5,1)$ | 5.852982 | 5.754966 | 5.785973 | 5.882043 | 5.819792 | 5.778357 | 5.743501 | 5.528077 | 5.676859 | 6.119393 | 5.716782 |
| $(5,2)$ | 7.005873 | 6.85517 | 6.837399 | 7.054765 | 6.958546 | 6.844823 | 6.934199 | 6.807395 | 6.89077 | 7.170421 | 6.754797 |
| $(5,3)$ | 7.650707 | 7.512163 | 7.482027 | 7.693078 | 7.608942 | 7.493288 | 7.606949 | 7.522834 | 7.577746 | 7.772003 | 7.406035 |
| $(5,4)$ | 8.097609 | 7.974628 | 7.940566 | 8.134081 | 8.061383 | 7.953017 | 8.066096 | 8.001332 | 8.043557 | 8.195279 | 7.872935 |
| $(5,5)$ | 8.439311 | 8.329503 | 8.294203 | 8.471371 | 8.407435 | 8.306997 | 8.414374 | 8.360578 | 8.395666 | 8.522128 | 8.23401 |

**Table S4:** Entropy values for PHOTH-G$(m, n)$ at $k = 2$

| $k=2$ | $I_{M_2\Re M_1}$ | $I_{M_2\Re M_2}$ | $I_{M_2\Re F}$ | $I_{M_2\Re GA}$ | $I_{M_2\Re BM}$ | $I_{M_2\Re TM}$ | $I_{M_2\Re HG}$ | $I_{M_2\Re HTM}$ | $I_{M_2\Re HBM}$ | $I_{M_2\Re BMG}$ | $I_{M_2\Re BMH}$ |
|---|---|---|---|---|---|---|---|---|---|---|---|
| $(1,1)$ | 3.448604 | 3.415905 | 3.42342 | 3.459362 | 3.432598 | 3.421169 | 3.403113 | 3.327592 | 3.364904 | 4.343866 | 3.39514 |
| $(1,2)$ | 4.624092 | 4.564796 | 4.564827 | 4.643791 | 4.596447 | 4.565044 | 4.574804 | 4.494265 | 4.536201 | 5.363002 | 4.516466 |
| $(1,3)$ | 5.267551 | 5.212849 | 5.20947 | 5.285339 | 5.242548 | 5.210807 | 5.229399 | 5.16527 | 5.199248 | 5.954837 | 5.164543 |
| $(1,4)$ | 5.711837 | 5.662513 | 5.657205 | 5.727857 | 5.689515 | 5.659174 | 5.679553 | 5.62389 | 5.653801 | 6.373391 | 5.616944 |
| $(1,5)$ | 6.051314 | 6.006289 | 5.999745 | 6.066027 | 6.031057 | 6.002119 | 6.022535 | 5.971928 | 5.999423 | 6.697454 | 5.96342 |
| $(2,1)$ | 4.511942 | 4.475454 | 4.482635 | 4.523487 | 4.494496 | 4.480553 | 4.470964 | 4.401303 | 4.437197 | 5.345718 | 4.450355 |
| $(2,2)$ | 5.682589 | 5.627887 | 5.624507 | 5.700376 | 5.657585 | 5.625844 | 5.644437 | 5.580308 | 5.614285 | 6.369875 | 5.579581 |
| $(2,3)$ | 6.324434 | 6.275959 | 6.270077 | 6.339795 | 6.302688 | 6.272221 | 6.296462 | 6.248422 | 6.274027 | 6.963248 | 6.229768 |
| $(2,4)$ | 6.767912 | 6.725324 | 6.718476 | 6.781309 | 6.748989 | 6.72093 | 6.745206 | 6.705421 | 6.726778 | 7.382548 | 6.683023 |
| $(2,5)$ | 7.106905 | 7.068807 | 7.061489 | 7.118893 | 7.090073 | 7.064092 | 7.087324 | 7.052468 | 7.071302 | 7.707051 | 7.029927 |
| $(3,1)$ | 5.118017 | 5.082495 | 5.088764 | 5.129209 | 5.101167 | 5.086985 | 5.080006 | 5.013997 | 5.048692 | 5.931392 | 5.057295 |
| $(3,2)$ | 6.28705 | 6.23569 | 6.231043 | 6.303718 | 6.26373 | 6.232796 | 6.252809 | 6.194324 | 6.225587 | 6.957176 | 6.188977 |
| $(3,3)$ | 6.928357 | 6.883589 | 6.877039 | 6.942462 | 6.908402 | 6.879398 | 6.903899 | 6.861364 | 6.884133 | 7.551048 | 6.839752 |
| $(3,4)$ | 7.371567 | 7.332756 | 7.325728 | 7.383658 | 7.354431 | 7.328231 | 7.352154 | 7.3178 | 7.336264 | 7.970591 | 7.293219 |
| $(3,5)$ | 7.710398 | 7.676081 | 7.668941 | 7.721057 | 7.695332 | 7.671473 | 7.693977 | 7.66451 | 7.680416 | 8.295237 | 7.640216 |
| $(4,1)$ | 5.54361 | 5.509028 | 5.514669 | 5.554516 | 5.527269 | 5.513094 | 5.50725 | 5.443244 | 5.477265 | 6.346803 | 5.48408 |
| $(4,2)$ | 6.711837 | 6.662513 | 6.657205 | 6.727857 | 6.689515 | 6.659174 | 6.679553 | 6.62389 | 6.653801 | 7.373391 | 6.616944 |
| $(4,3)$ | 7.352875 | 7.310286 | 7.303439 | 7.366271 | 7.333952 | 7.305893 | 7.330169 | 7.290383 | 7.311741 | 7.967511 | 7.267986 |
| $(4,4)$ | 7.79595 | 7.759334 | 7.752286 | 7.807311 | 7.779835 | 7.754791 | 7.778178 | 7.746538 | 7.763561 | 8.387173 | 7.721542 |
| $(4,5)$ | 8.134701 | 8.102567 | 8.095609 | 8.144619 | 8.120638 | 8.098073 | 8.119853 | 8.09308 | 8.107529 | 8.71189 | 8.068574 |
| $(5,1)$ | 5.871872 | 5.838003 | 5.843212 | 5.882571 | 5.855906 | 5.841775 | 5.836544 | 5.773785 | 5.807382 | 6.66896 | 5.813312 |
| $(5,2)$ | 7.039615 | 6.991632 | 6.985919 | 7.05522 | 7.017943 | 6.988021 | 7.008503 | 6.954529 | 6.983632 | 7.696029 | 6.946869 |
| $(5,3)$ | 7.680491 | 7.639317 | 7.632303 | 7.693437 | 7.662232 | 7.63481 | 7.658831 | 7.620693 | 7.641206 | 8.290296 | 7.598061 |
| $(5,4)$ | 8.123486 | 8.088287 | 8.081251 | 8.134384 | 8.108024 | 8.083748 | 8.106693 | 8.076679 | 8.092841 | 8.71003 | 8.051665 |
| $(5,5)$ | 8.462188 | 8.43146 | 8.424643 | 8.471638 | 8.448767 | 8.427055 | 8.448279 | 8.42312 | 8.436697 | 9.034789 | 8.398714 |

**Table S5:** Entropy value for PHOTH-G$(m, n)$ at $k = 3$

| $k=3$ | $I_{M_3\Re M_1}$ | $I_{M_3\Re M_2}$ | $I_{M_3\Re F}$ | $I_{M_3\Re GA}$ | $I_{M_3\Re BM}$ | $I_{M_3\Re TM}$ | $I_{M_3\Re HG}$ | $I_{M_3\Re HTM}$ | $I_{M_3\Re HBM}$ | $I_{M_3\Re BMG}$ | $I_{M_3\Re BMH}$ |
|---|---|---|---|---|---|---|---|---|---|---|---|
| (1,1) | 3.337175 | 2.993818 | 2.987343 | 3.456075 | 3.213711 | 2.99853 | 3.194465 | 2.948489 | 3.104308 | 3.727843 | 2.786628 |
| (1,2) | 4.539473 | 4.283617 | 4.375084 | 4.640725 | 4.426481 | 4.353822 | 4.093493 | 3.530482 | 3.868693 | 5.27042 | 4.21024 |
| (1,3) | 5.20741 | 5.011167 | 5.098544 | 5.282344 | 5.118729 | 5.077433 | 4.713688 | 4.010565 | 4.439442 | 6.000002 | 4.963926 |
| (1,4) | 5.663946 | 5.495032 | 5.579798 | 5.724898 | 5.58707 | 5.559042 | 5.178958 | 4.415463 | 4.888214 | 6.482404 | 5.458444 |
| (1,5) | 6.01064 | 5.857285 | 5.940426 | 6.063088 | 5.940642 | 5.919927 | 5.547502 | 4.763116 | 5.254384 | 6.843333 | 5.826472 |
| (2,1) | 4.424268 | 4.14064 | 4.202449 | 4.519832 | 4.318388 | 4.194751 | 4.176296 | 3.757198 | 4.032224 | 4.894786 | 4.013468 |
| (2,2) | 5.622448 | 5.426204 | 5.513581 | 5.697382 | 5.533767 | 5.492471 | 5.128726 | 4.425603 | 4.854479 | 6.415039 | 5.378963 |
| (2,3) | 6.282815 | 6.14121 | 6.210674 | 6.337211 | 6.217121 | 6.193324 | 5.773089 | 4.963705 | 5.458325 | 7.140457 | 6.111274 |
| (2,4) | 6.735491 | 6.619459 | 6.680198 | 6.778951 | 6.681161 | 6.664835 | 6.251081 | 5.409088 | 5.927808 | 7.62113 | 6.596745 |
| (2,5) | 7.079886 | 6.978554 | 7.034223 | 7.116677 | 7.032236 | 7.020048 | 6.627014 | 5.785865 | 6.307809 | 7.981114 | 6.959795 |
| (3,1) | 5.041223 | 4.778148 | 4.857805 | 5.125587 | 4.942371 | 4.845265 | 4.77099 | 4.28901 | 4.610375 | 5.531369 | 4.672226 |
| (3,2) | 6.2351 | 6.057271 | 6.142926 | 6.300747 | 6.154333 | 6.122039 | 5.743845 | 4.998195 | 5.457321 | 7.045249 | 6.017283 |
| (3,3) | 6.892892 | 6.768482 | 6.832095 | 6.940028 | 6.834802 | 6.816069 | 6.396703 | 5.562226 | 6.074639 | 7.769491 | 6.743453 |
| (3,4) | 7.344289 | 7.245045 | 7.298061 | 7.381528 | 7.297474 | 7.284535 | 6.878811 | 6.024819 | 6.552217 | 8.24968 | 7.226308 |
| (3,5) | 7.687928 | 7.603193 | 7.650073 | 7.719119 | 7.64776 | 7.638032 | 7.256935 | 6.413402 | 6.937427 | 8.609401 | 7.587915 |
| (4,1) | 5.47254 | 5.219536 | 5.307378 | 5.550937 | 5.377247 | 5.292629 | 5.195437 | 4.682649 | 5.027659 | 5.971542 | 5.123389 |
| (4,2) | 6.663946 | 6.495032 | 6.579798 | 6.724898 | 6.58707 | 6.559042 | 6.178958 | 5.415463 | 5.888214 | 7.482404 | 6.458444 |
| (4,3) | 7.320454 | 7.204421 | 7.26516 | 7.363914 | 7.266123 | 7.249797 | 6.836043 | 5.99405 | 6.512771 | 8.206093 | 7.181707 |
| (4,4) | 7.771216 | 7.680174 | 7.729411 | 7.805299 | 7.728128 | 7.716799 | 7.320097 | 6.466025 | 6.994597 | 8.686054 | 7.663321 |
| (4,5) | 8.11448 | 8.037869 | 8.08045 | 8.142826 | 8.078029 | 8.069469 | 7.699192 | 6.860857 | 7.38248 | 9.045651 | 8.024242 |
| (5,1) | 5.804303 | 5.557258 | 5.649793 | 5.879026 | 5.711146 | 5.633781 | 5.524766 | 4.994196 | 5.353251 | 6.308344 | 5.466723 |
| (5,2) | 6.994143 | 6.830485 | 6.914712 | 7.052268 | 6.919578 | 6.894039 | 6.514813 | 5.742398 | 6.222411 | 7.817447 | 6.79587 |
| (5,3) | 7.649882 | 7.538805 | 7.597839 | 7.691126 | 7.597792 | 7.582872 | 7.174404 | 6.330269 | 6.851467 | 8.540813 | 7.517438 |
| (5,4) | 8.100266 | 8.014083 | 8.06108 | 8.132444 | 8.059401 | 8.049013 | 7.659569 | 6.808109 | 7.335895 | 9.020642 | 7.998328 |
| (5,5) | 8.443307 | 8.371511 | 8.411546 | 8.469933 | 8.409074 | 8.401197 | 8.039192 | 7.20677 | 7.72539 | 9.380168 | 8.358848 |

**Table S6:** Entropy value for S-G$(m, n)$ at $k = 1$

| $k=1$ | $I_{M_1\Re M_1}$ | $I_{M_1\Re M_2}$ | $I_{M_1\Re F}$ | $I_{M_1\Re GA}$ | $I_{M_1\Re BM}$ | $I_{M_1\Re TM}$ | $I_{M_1\Re HG}$ | $I_{M_1\Re HTM}$ | $I_{M_1\Re HBM}$ | $I_{M_1\Re BMG}$ | $I_{M_1\Re BMH}$ |
|---|---|---|---|---|---|---|---|---|---|---|---|
| (1, 1) | 3.139572 | 3.043856 | 3.080099 | 3.16931 | 3.105115 | 3.070375 | 2.999375 | 2.751225 | 2.91621 | 3.482374 | 3.01268 |
| (1,2) | 4.270942 | 4.121928 | 4.131282 | 4.321333 | 4.221252 | 4.130241 | 4.151808 | 3.953138 | 4.084499 | 4.504863 | 4.044234 |
| (1,3) | 4.905617 | 4.763178 | 4.756019 | 4.953608 | 4.859791 | 4.760152 | 4.812498 | 4.649109 | 4.757978 | 5.097306 | 4.67711 |
| (1,4) | 5.346756 | 5.214997 | 5.197234 | 5.391733 | 5.305162 | 5.20465 | 5.266159 | 5.119308 | 5.217795 | 5.51608 | 5.128047 |
| (1,5) | 5.684763 | 5.562332 | 5.537262 | 5.727339 | 5.646547 | 5.546926 | 5.611315 | 5.473968 | 5.566529 | 5.840249 | 5.476393 |
| (2,1) | 4.202134 | 4.069631 | 4.095556 | 4.247322 | 4.155903 | 4.089217 | 4.050976 | 3.800518 | 3.9661 | 4.493626 | 4.013245 |
| (2,2) | 5.337678 | 5.169925 | 5.161584 | 5.391816 | 5.283892 | 5.165993 | 5.254751 | 5.116281 | 5.206109 | 5.525308 | 5.066508 |
| (2,3) | 5.974172 | 5.821276 | 5.79869 | 6.021913 | 5.926969 | 5.807587 | 5.916506 | 5.812138 | 5.880148 | 6.12069 | 5.713679 |
| (2,4) | 6.416316 | 6.278867 | 6.248519 | 6.459002 | 6.374735 | 6.259866 | 6.369802 | 6.280992 | 6.339172 | 6.540912 | 6.174247 |
| (2,5) | 6.754959 | 6.629937 | 6.594712 | 6.794001 | 6.7176 | 6.6076 | 6.714549 | 6.634612 | 6.687217 | 6.865942 | 6.529444 |
| (3,1) | 4.809304 | 4.668522 | 4.689778 | 4.857378 | 4.760857 | 4.684945 | 4.666651 | 4.432091 | 4.586951 | 5.082336 | 4.604335 |
| (3,2) | 5.946439 | 5.777363 | 5.763809 | 5.99954 | 5.893069 | 5.769821 | 5.87651 | 5.75719 | 5.834206 | 6.117021 | 5.667523 |
| (3,3) | 6.583622 | 6.432599 | 6.407085 | 6.628963 | 6.537758 | 6.416845 | 6.537689 | 6.451605 | 6.507289 | 6.713367 | 6.321187 |
| (3,4) | 7.026146 | 6.89241 | 6.861172 | 7.065731 | 6.986373 | 6.872729 | 6.990557 | 6.919606 | 6.965691 | 7.134063 | 6.786136 |
| (3,5) | 7.365029 | 7.244916 | 7.210411 | 7.400543 | 7.329762 | 7.222993 | 7.335018 | 7.272697 | 7.313337 | 7.459376 | 7.144453 |
| (4,1) | 5.235713 | 5.091744 | 5.110373 | 5.284801 | 5.186535 | 5.106382 | 5.098354 | 4.872523 | 5.021594 | 5.499247 | 5.023869 |
| (4,2) | 6.373686 | 6.20511 | 6.189164 | 6.425828 | 6.320913 | 6.195909 | 6.310131 | 6.200045 | 6.270936 | 6.535423 | 6.092693 |
| (4,3) | 7.01123 | 6.862402 | 6.836043 | 7.054922 | 6.966428 | 6.846028 | 6.970927 | 6.893632 | 6.943437 | 7.132247 | 6.750041 |
| (4,4) | 7.453953 | 7.323389 | 7.292617 | 7.491533 | 7.415477 | 7.303967 | 7.423549 | 7.36118 | 7.401496 | 7.553179 | 7.217478 |
| (4,5) | 7.792961 | 7.676656 | 7.643635 | 7.826254 | 7.759135 | 7.655673 | 7.767853 | 7.713996 | 7.748929 | 7.878633 | 7.577569 |
| (5,1) | 5.564552 | 5.419007 | 5.435956 | 5.61411 | 5.515064 | 5.432503 | 5.430606 | 5.210142 | 5.35568 | 5.822298 | 5.349005 |
| (5,2) | 6.703041 | 6.535175 | 6.517877 | 6.754465 | 6.65076 | 6.525034 | 6.643245 | 6.538582 | 6.605891 | 6.859365 | 6.421454 |
| (5,3) | 7.340807 | 7.19374 | 7.167107 | 7.383364 | 7.296776 | 7.177147 | 7.303786 | 7.23165 | 7.278017 | 7.456476 | 7.081173 |
| (5,4) | 7.783652 | 7.655455 | 7.625304 | 7.819883 | 7.746089 | 7.63641 | 7.756254 | 7.698921 | 7.735864 | 7.877549 | 7.550211 |
| (5,5) | 8.122738 | 8.009192 | 7.977484 | 8.15455 | 8.08991 | 7.989046 | 8.10046 | 8.05157 | 8.083165 | 8.203087 | 7.911444 |

**Table S7:** Entropy value for S-G$(m, n)$ at $k = 2$

| $k=2$ | $I_{M_2\Re M_1}$ | $I_{M_2\Re M_2}$ | $I_{M_2\Re F}$ | $I_{M_2\Re GA}$ | $I_{M_2\Re BM}$ | $I_{M_2\Re TM}$ | $I_{M_2\Re HG}$ | $I_{M_2\Re HTM}$ | $I_{M_2\Re HBM}$ | $I_{M_2\Re BMG}$ | $I_{M_2\Re BMH}$ |
|---|---|---|---|---|---|---|---|---|---|---|---|
| $(1,1)$ | 3.158345 | 3.122556 | 3.130322 | 3.169851 | 3.14103 | 3.128027 | 3.114074 | 3.040233 | 3.077375 | 4.022809 | 3.098946 |
| $(1,2)$ | 4.304585 | 4.252847 | 4.252389 | 4.321856 | 4.280546 | 4.252786 | 4.259835 | 4.183658 | 4.224331 | 5.039017 | 4.210241 |
| $(1,3)$ | 4.938295 | 4.890921 | 4.8869 | 4.954125 | 4.916625 | 4.888459 | 4.901667 | 4.837515 | 4.872436 | 5.629719 | 4.848904 |
| $(1,4)$ | 5.377705 | 5.334661 | 5.328666 | 5.392247 | 5.358167 | 5.330868 | 5.345506 | 5.287823 | 5.319649 | 6.047683 | 5.294841 |
| $(1,5)$ | 5.714258 | 5.674548 | 5.667301 | 5.72785 | 5.696312 | 5.66991 | 5.684754 | 5.631009 | 5.660955 | 6.371384 | 5.636762 |
| $(2,1)$ | 4.231465 | 4.182753 | 4.186813 | 4.247854 | 4.208321 | 4.185733 | 4.179459 | 4.09189 | 4.13775 | 5.030711 | 4.146573 |
| $(2,2)$ | 5.37468 | 5.320122 | 5.316178 | 5.392256 | 5.349851 | 5.317695 | 5.338724 | 5.278335 | 5.310265 | 6.050202 | 5.271123 |
| $(2,3)$ | 6.00738 | 5.96032 | 5.954135 | 6.022312 | 5.986305 | 5.956379 | 5.980406 | 5.933797 | 5.958722 | 6.641917 | 5.915101 |
| $(2,4)$ | 6.446287 | 6.405053 | 6.397866 | 6.459379 | 6.427967 | 6.400435 | 6.423809 | 6.384105 | 6.405551 | 7.060374 | 6.363904 |
| $(2,5)$ | 6.782536 | 6.74551 | 6.737766 | 6.794365 | 6.766162 | 6.740515 | 6.762712 | 6.727116 | 6.7465 | 7.384366 | 6.707615 |
| $(3,1)$ | 4.840801 | 4.789898 | 4.792552 | 4.857908 | 4.816789 | 4.791932 | 4.790184 | 4.704931 | 4.749849 | 5.618399 | 4.750767 |
| $(3,2)$ | 5.983007 | 5.929531 | 5.924816 | 5.999944 | 5.958853 | 5.926577 | 5.950987 | 5.897165 | 5.925527 | 6.638944 | 5.880141 |
| $(3,3)$ | 6.615371 | 6.570385 | 6.56413 | 6.629309 | 6.595379 | 6.566382 | 6.592263 | 6.552343 | 6.573537 | 7.230984 | 6.526051 |
| $(3,4)$ | 7.05411 | 7.01542 | 7.008633 | 7.066045 | 7.037055 | 7.011051 | 7.035393 | 7.002371 | 7.02004 | 7.649598 | 6.975895 |
| $(3,5)$ | 7.390258 | 7.356047 | 7.349028 | 7.400838 | 7.375249 | 7.351517 | 7.374117 | 7.345184 | 7.360774 | 7.973684 | 7.320258 |
| $(4,1)$ | 5.268025 | 5.216458 | 5.218372 | 5.28533 | 5.243789 | 5.217994 | 5.218553 | 5.135126 | 5.179215 | 6.034816 | 5.176134 |
| $(4,2)$ | 6.409727 | 6.357199 | 6.352185 | 6.426212 | 6.386093 | 6.354038 | 6.379766 | 6.329331 | 6.355863 | 7.055879 | 6.308004 |
| $(4,3)$ | 6.358501 | 6.30743 | 6.302469 | 6.374978 | 6.335376 | 6.304321 | 6.32563 | 6.269494 | 6.299463 | 7.021356 | 6.260488 |
| $(4,4)$ | 7.480577 | 7.443549 | 7.437134 | 7.491814 | 7.464321 | 7.439418 | 7.463773 | 7.434136 | 7.449905 | 8.066772 | 7.405296 |
| $(4,5)$ | 7.816676 | 7.784258 | 7.777794 | 7.826512 | 7.802512 | 7.780086 | 7.8024 | 7.776836 | 7.79052 | 8.390904 | 7.749995 |
| $(5,1)$ | 5.597265 | 5.545442 | 5.546901 | 5.614637 | 5.572964 | 5.546671 | 5.548598 | 5.466432 | 5.509936 | 6.357577 | 5.5045 |
| $(5,2)$ | 6.738664 | 6.686841 | 6.681676 | 6.754836 | 6.715402 | 6.683575 | 6.709958 | 6.661578 | 6.687004 | 7.378949 | 6.637896 |
| $(5,3)$ | 7.37076 | 7.328198 | 7.32216 | 7.383663 | 7.351966 | 7.324324 | 7.350827 | 7.316362 | 7.334537 | 7.971245 | 7.285417 |
| $(5,4)$ | 7.809364 | 7.77346 | 7.767328 | 7.820143 | 7.793641 | 7.76951 | 7.793711 | 7.766111 | 7.78074 | 8.389984 | 7.736127 |
| $(5,5)$ | 8.145432 | 8.114216 | 8.108152 | 8.154785 | 8.131829 | 8.110302 | 8.132277 | 8.108739 | 8.121281 | 8.714143 | 8.081032 |

**Table S8:** Entropy value for S-G$(m, n)$ at $k = 3$

| $k=3$ | $I_{M_3\Re M_1}$ | $I_{M_3\Re M_2}$ | $I_{M_3\Re F}$ | $I_{M_3\Re GA}$ | $I_{M_3\Re BM}$ | $I_{M_3\Re TM}$ | $I_{M_3\Re HG}$ | $I_{M_3\Re HTM}$ | $I_{M_3\Re HBM}$ | $I_{M_3\Re BMG}$ | $I_{M_3\Re BMH}$ |
|---|---|---|---|---|---|---|---|---|---|---|---|
| $(1,1)$ | 3.056565 | 2.741901 | 2.77264 | 3.166288 | 2.940835 | 2.773461 | 2.856603 | 2.528253 | 2.73955 | 3.491795 | 2.578012 |
| $(1,2)$ | 4.23127 | 3.981119 | 4.089776 | 4.318423 | 4.123428 | 4.065036 | 3.814171 | 3.204795 | 3.586624 | 4.933363 | 3.913676 |
| $(1,3)$ | 4.884026 | 4.679791 | 4.786094 | 4.950739 | 4.794069 | 4.760964 | 4.449398 | 3.741404 | 4.191345 | 5.640782 | 4.63375 |
| $(1,4)$ | 5.333136 | 5.150891 | 5.255156 | 5.388885 | 5.252282 | 5.23017 | 4.916448 | 4.180868 | 4.653807 | 6.113481 | 5.113902 |
| $(1,5)$ | 5.675471 | 5.505994 | 5.608894 | 5.724503 | 5.600033 | 5.58406 | 5.282262 | 4.549782 | 5.024368 | 6.468966 | 5.473993 |
| $(2,1)$ | 4.134469 | 3.819676 | 3.919622 | 4.244353 | 4.006028 | 3.899458 | 3.813726 | 3.357947 | 3.643795 | 4.728635 | 3.709365 |
| $(2,2)$ | 5.318334 | 5.136179 | 5.217496 | 5.3894 | 5.235239 | 5.197636 | 4.8093 | 4.080746 | 4.522858 | 6.130602 | 5.093292 |
| $(2,3)$ | 5.967775 | 5.830351 | 5.899406 | 6.019734 | 5.903991 | 5.882132 | 5.465935 | 4.65025 | 5.150652 | 6.828951 | 5.8019 |
| $(2,4)$ | 6.414865 | 6.298358 | 6.361469 | 6.456948 | 6.360471 | 6.34553 | 5.946715 | 5.114213 | 5.630397 | 7.297636 | 6.276117 |
| $(2,5)$ | 6.755915 | 6.651477 | 6.711118 | 6.792024 | 6.707033 | 6.695978 | 6.322083 | 5.502032 | 6.014342 | 7.650856 | 6.632689 |
| $(3,1)$ | 4.750542 | 4.455204 | 4.560916 | 4.854429 | 4.627335 | 4.538355 | 4.392209 | 3.887642 | 4.203123 | 5.384977 | 4.360825 |
| $(3,2)$ | 5.933206 | 5.774355 | 5.845464 | 5.997337 | 5.859707 | 5.827819 | 5.403689 | 4.631395 | 5.096859 | 6.776092 | 5.738173 |
| $(3,3)$ | 6.581174 | 6.466019 | 6.522508 | 6.627087 | 6.526974 | 6.508181 | 6.069262 | 5.215664 | 5.735302 | 7.471912 | 6.44247 |
| $(3,4)$ | 7.027474 | 6.932727 | 6.982271 | 7.064028 | 6.982612 | 6.969593 | 6.555765 | 5.690544 | 6.223059 | 7.939473 | 6.914644 |
| $(3,5)$ | 7.368043 | 7.285072 | 7.330587 | 7.398947 | 7.328654 | 7.318884 | 6.93511 | 6.086737 | 6.613226 | 8.292058 | 7.270036 |
| $(4,1)$ | 5.181658 | 4.897157 | 5.004427 | 5.281862 | 5.061838 | 4.981061 | 4.806146 | 4.276102 | 4.607342 | 5.834476 | 4.810107 |
| $(4,2)$ | 6.363259 | 6.215883 | 6.281908 | 6.423738 | 6.294628 | 6.265409 | 5.826366 | 5.031806 | 5.509494 | 7.220523 | 6.182813 |
| $(4,3)$ | 7.010453 | 6.906234 | 6.956556 | 7.053209 | 6.961076 | 6.943713 | 6.496844 | 5.624458 | 6.154063 | 7.915156 | 6.884977 |
| $(4,4)$ | 7.456348 | 7.372283 | 7.415191 | 7.49002 | 7.416272 | 7.404145 | 6.986487 | 6.105513 | 6.646438 | 8.382188 | 7.356151 |
| $(4,5)$ | 7.796671 | 7.724238 | 7.762854 | 7.824863 | 7.762045 | 7.752866 | 7.368013 | 6.506429 | 7.040198 | 8.734475 | 7.710968 |
| $(5,1)$ | 5.513364 | 5.235576 | 5.343466 | 5.611176 | 5.395754 | 5.319715 | 5.128219 | 4.58259 | 4.923576 | 6.176708 | 5.152725 |
| $(5,2)$ | 6.694206 | 6.553639 | 6.616634 | 6.752446 | 6.628504 | 6.600831 | 6.153965 | 5.346018 | 5.831185 | 7.559821 | 6.522366 |
| $(5,3)$ | 7.340926 | 7.243192 | 7.289861 | 7.381752 | 7.294442 | 7.277908 | 6.827543 | 5.944072 | 6.479733 | 8.253765 | 7.223262 |
| $(5,4)$ | 7.786575 | 7.708845 | 7.747826 | 7.818487 | 7.749369 | 7.737757 | 7.319168 | 6.429098 | 6.975112 | 8.72049 | 7.693846 |
| $(5,5)$ | 8.12675 | 8.060567 | 8.095102 | 8.153286 | 8.094978 | 8.08614 | 7.702071 | 6.833046 | 7.37121 | 9.072604 | 8.048323 |

**Table S9:** Entropy value for PH-G$(m, n)$ at $k = 1$

| $k=1$ | $I_{M_1\Re M_1}$ | $I_{M_1\Re M_2}$ | $I_{M_1\Re F}$ | $I_{M_1\Re GA}$ | $I_{M_1\Re BM}$ | $I_{M_1\Re TM}$ | $I_{M_1\Re HG}$ | $I_{M_1\Re HTM}$ | $I_{M_1\Re HBM}$ | $I_{M_1\Re BMG}$ | $I_{M_1\Re BMH}$ |
|---|---|---|---|---|---|---|---|---|---|---|---|
| $(1,1)$ | 3.139572 | 3.043856 | 3.080099 | 3.16931 | 3.105115 | 3.070375 | 2.999375 | 2.751225 | 2.91621 | 3.482374 | 3.01268 |
| $(1,2)$ | 4.219039 | 4.123939 | 4.152305 | 4.24734 | 4.187119 | 4.145504 | 4.114216 | 3.903414 | 4.049905 | 4.474645 | 4.085631 |
| $(1,3)$ | 4.832105 | 4.750409 | 4.768108 | 4.857438 | 4.805337 | 4.764438 | 4.740141 | 4.541969 | 4.682211 | 5.05718 | 4.713405 |
| $(1,4)$ | 5.261514 | 5.189857 | 5.200073 | 5.284887 | 5.23829 | 5.198513 | 5.175819 | 4.983799 | 5.121006 | 5.471032 | 5.154635 |
| $(1,5)$ | 5.59217 | 5.527748 | 5.532677 | 5.614213 | 5.571406 | 5.532579 | 5.510155 | 5.321763 | 5.457183 | 5.792257 | 5.494042 |
| $(2,1)$ | 4.270942 | 4.121928 | 4.131282 | 4.321333 | 4.221252 | 4.130241 | 4.151808 | 3.953138 | 4.084499 | 4.504863 | 4.044234 |
| $(2,2)$ | 5.346756 | 5.214997 | 5.197234 | 5.391733 | 5.305162 | 5.20465 | 5.266159 | 5.119308 | 5.217795 | 5.51608 | 5.128047 |
| $(2,3)$ | 5.958695 | 5.843856 | 5.813465 | 5.99942 | 5.923109 | 5.824758 | 5.889824 | 5.758636 | 5.847373 | 6.104776 | 5.759385 |
| $(2,4)$ | 6.38756 | 6.283906 | 6.246302 | 6.425686 | 6.35571 | 6.259796 | 6.324174 | 6.200483 | 6.28458 | 6.52168 | 6.202324 |
| $(2,5)$ | 6.717896 | 6.621928 | 6.579671 | 6.75431 | 6.688537 | 6.594579 | 6.657664 | 6.538365 | 6.619751 | 6.844727 | 6.542718 |
| $(3,1)$ | 4.905617 | 4.763178 | 4.756019 | 4.953608 | 4.859791 | 4.760152 | 4.812498 | 4.649109 | 4.757978 | 5.097306 | 4.67711 |
| $(3,2)$ | 5.980377 | 5.857645 | 5.824678 | 6.021843 | 5.942811 | 5.836853 | 5.922275 | 5.811663 | 5.885604 | 6.114594 | 5.764605 |
| $(3,3)$ | 6.592002 | 6.485848 | 6.443434 | 6.628858 | 6.560063 | 6.458567 | 6.544392 | 6.44978 | 6.513341 | 6.705275 | 6.396995 |
| $(3,4)$ | 7.020718 | 6.92526 | 6.877964 | 7.054798 | 6.992224 | 6.894628 | 6.977978 | 6.891035 | 6.94965 | 7.123163 | 6.840388 |
| $(3,5)$ | 7.350967 | 7.262783 | 7.212507 | 7.383228 | 7.324755 | 7.230107 | 7.311014 | 7.228568 | 7.284292 | 7.446799 | 7.181024 |
| $(4,1)$ | 5.346756 | 5.214997 | 5.197234 | 5.391733 | 5.305162 | 5.20465 | 5.266159 | 5.119308 | 5.217795 | 5.51608 | 5.128047 |
| $(4,2)$ | 6.42103 | 6.309035 | 6.268965 | 6.458944 | 6.387315 | 6.283379 | 6.3736 | 6.280022 | 6.342616 | 6.536361 | 6.217228 |
| $(4,3)$ | 7.032513 | 6.936408 | 6.889839 | 7.065644 | 7.004056 | 6.906311 | 6.994955 | 6.917555 | 6.969471 | 7.128021 | 6.850034 |
| $(4,4)$ | 7.461162 | 7.375222 | 7.325721 | 7.49143 | 7.435912 | 7.343124 | 7.428168 | 7.358528 | 7.405353 | 7.546395 | 7.293582 |
| $(4,5)$ | 7.791372 | 7.712319 | 7.661184 | 7.81977 | 7.768243 | 7.679107 | 7.760982 | 7.695895 | 7.739744 | 7.870322 | 7.634289 |
| $(5,1)$ | 5.684763 | 5.562332 | 5.537262 | 5.727339 | 5.646547 | 5.546926 | 5.611315 | 5.473968 | 5.566529 | 5.840249 | 5.476393 |
| $(5,2)$ | 6.758759 | 6.655623 | 6.611565 | 6.793953 | 6.72802 | 6.627241 | 6.717364 | 6.633598 | 6.689705 | 6.862311 | 6.566488 |
| $(5,3)$ | 7.370162 | 7.282289 | 7.234076 | 7.400469 | 7.344396 | 7.251077 | 7.338271 | 7.270793 | 7.316048 | 7.454555 | 7.199485 |
| $(5,4)$ | 7.798774 | 7.72063 | 7.670986 | 7.826167 | 7.776037 | 7.688442 | 7.771265 | 7.711603 | 7.751682 | 7.87322 | 7.64309 |
| $(5,5)$ | 8.128963 | 8.057398 | 8.007145 | 8.154454 | 8.108229 | 8.024792 | 8.10395 | 8.048875 | 8.085927 | 8.19732 | 7.983816 |

**Table S10:** Entropy value for PH-G$(m, n)$ at $k = 2$

| $k=2$ | $I_{M_2\Re M_1}$ | $I_{M_2\Re M_2}$ | $I_{M_2\Re F}$ | $I_{M_2\Re GA}$ | $I_{M_2\Re BM}$ | $I_{M_2\Re TM}$ | $I_{M_2\Re HG}$ | $I_{M_2\Re HTM}$ | $I_{M_2\Re HBM}$ | $I_{M_2\Re BMG}$ | $I_{M_2\Re BMH}$ |
|---|---|---|---|---|---|---|---|---|---|---|---|
| $(1,1)$ | 3.158345 | 3.122556 | 3.130322 | 3.169851 | 3.14103 | 3.128027 | 3.114074 | 3.040233 | 3.077375 | 4.022809 | 3.098946 |
| $(1,2)$ | 4.237517 | 4.204936 | 4.20941 | 4.247857 | 4.22221 | 4.208207 | 4.203787 | 4.142951 | 4.175892 | 5.025455 | 4.180784 |
| $(1,3)$ | 4.84887 | 4.821291 | 4.823167 | 4.857916 | 4.836027 | 4.82278 | 4.81982 | 4.764576 | 4.795588 | 5.61144 | 4.799702 |
| $(1,4)$ | 5.277103 | 5.252847 | 5.253094 | 5.285341 | 5.265849 | 5.253213 | 5.250479 | 5.198124 | 5.228134 | 6.027014 | 5.233189 |
| $(1,5)$ | 5.606948 | 5.584963 | 5.584119 | 5.614651 | 5.596765 | 5.584574 | 5.581796 | 5.531193 | 5.560593 | 6.349272 | 5.56669 |
| $(2,1)$ | 4.304585 | 4.252847 | 4.252389 | 4.321856 | 4.280546 | 4.252786 | 4.259835 | 4.183658 | 4.224331 | 5.039017 | 4.210241 |
| $(2,2)$ | 5.377705 | 5.334661 | 5.328666 | 5.392247 | 5.358167 | 5.330868 | 5.345506 | 5.287823 | 5.319649 | 6.047683 | 5.294841 |
| $(2,3)$ | 5.987041 | 5.949875 | 5.941764 | 5.99993 | 5.970291 | 5.944654 | 5.959338 | 5.908228 | 5.936917 | 6.635607 | 5.91377 |
| $(2,4)$ | 6.414266 | 6.380649 | 6.371423 | 6.426195 | 6.399162 | 6.374675 | 6.388805 | 6.340989 | 6.36811 | 7.052139 | 6.347038 |
| $(2,5)$ | 6.743506 | 6.712222 | 6.702308 | 6.754818 | 6.729472 | 6.705783 | 6.719383 | 6.673541 | 6.699723 | 7.374967 | 6.680329 |
| $(3,1)$ | 4.938295 | 4.890921 | 4.8869 | 4.954125 | 4.916625 | 4.888459 | 4.901667 | 4.837515 | 4.872436 | 5.629719 | 4.848904 |
| $(3,2)$ | 6.009398 | 5.970951 | 5.962763 | 6.022304 | 5.99219 | 5.965677 | 5.984256 | 5.938584 | 5.963892 | 6.640261 | 5.932901 |
| $(3,3)$ | 6.618061 | 6.585204 | 6.575847 | 6.629296 | 6.603455 | 6.579141 | 6.597011 | 6.557889 | 6.579821 | 7.22879 | 6.551211 |
| $(3,4)$ | 7.044949 | 7.01542 | 7.005526 | 7.055223 | 7.031862 | 7.008994 | 7.025938 | 6.990101 | 7.010345 | 7.64562 | 6.984071 |
| $(3,5)$ | 7.373987 | 7.34663 | 7.336432 | 7.383646 | 7.361882 | 7.339999 | 7.356193 | 7.322323 | 7.341559 | 7.968627 | 7.317082 |
| $(4,1)$ | 5.377705 | 5.334661 | 5.328666 | 5.392247 | 5.358167 | 5.330868 | 5.345506 | 5.287823 | 5.319649 | 6.047683 | 5.294841 |
| $(4,2)$ | 6.4478 | 6.413408 | 6.404457 | 6.459372 | 6.432515 | 6.407617 | 6.426486 | 6.387247 | 6.409098 | 7.059139 | 6.378097 |
| $(4,3)$ | 7.056127 | 7.027044 | 7.017586 | 7.066035 | 7.043288 | 7.020906 | 7.038697 | 7.005996 | 7.024353 | 7.647964 | 6.995925 |
| $(4,4)$ | 7.482847 | 7.456909 | 7.447303 | 7.491802 | 7.471431 | 7.450668 | 7.467353 | 7.437933 | 7.454545 | 8.06494 | 7.42849 |
| $(4,5)$ | 7.811785 | 7.787896 | 7.778237 | 7.820129 | 7.801288 | 7.781617 | 7.797446 | 7.769992 | 7.785561 | 8.388033 | 7.761305 |
| $(5,1)$ | 5.714258 | 5.674548 | 5.667301 | 5.72785 | 5.696312 | 5.66991 | 5.684754 | 5.631009 | 5.660955 | 6.371384 | 5.636762 |
| $(5,2)$ | 6.783747 | 6.752385 | 6.743098 | 6.79436 | 6.769866 | 6.746364 | 6.764762 | 6.72944 | 6.749195 | 7.383382 | 6.719404 |
| $(5,3)$ | 7.391872 | 7.365602 | 7.356273 | 7.400829 | 7.380323 | 7.359544 | 7.376647 | 7.347858 | 7.364051 | 7.972382 | 7.336878 |
| $(5,4)$ | 7.818491 | 7.795233 | 7.786023 | 7.826502 | 7.808297 | 7.789249 | 7.805142 | 7.779632 | 7.794045 | 8.389444 | 7.769231 |
| $(5,5)$ | 8.147368 | 8.126071 | 8.11698 | 8.154774 | 8.138049 | 8.120162 | 8.135138 | 8.111594 | 8.124943 | 8.712589 | 8.101906 |

**Table S11:** Entropy value for PH-G$(m, n)$ at $k = 3$

| $k=3$ | $I_{M_3\Re M_1}$ | $I_{M_3\Re M_2}$ | $I_{M_3\Re F}$ | $I_{M_3\Re GA}$ | $I_{M_3\Re BM}$ | $I_{M_3\Re TM}$ | $I_{M_3\Re HG}$ | $I_{M_3\Re HTM}$ | $I_{M_3\Re HBM}$ | $I_{M_3\Re BMG}$ | $I_{M_3\Re BMH}$ |
|---|---|---|---|---|---|---|---|---|---|---|---|
| $(1, 1)$ | 3.056565 | 2.741901 | 2.77264 | 3.166288 | 2.940835 | 2.773461 | 2.856603 | 2.528253 | 2.73955 | 3.491795 | 2.578012 |
| $(1, 2)$ | 4.026619 | 3.868016 | 3.890578 | 4.084246 | 3.970626 | 3.891073 | 3.787473 | 3.266865 | 3.630904 | 4.443426 | 3.788054 |
| $(1, 3)$ | 4.80172 | 4.587043 | 4.703468 | 4.854675 | 4.720531 | 4.681063 | 4.541054 | 3.941773 | 4.363681 | 5.33096 | 4.52515 |
| $(1, 4)$ | 5.237576 | 5.034152 | 5.158341 | 5.282257 | 5.160675 | 5.133871 | 5.003173 | 4.413395 | 4.835906 | 5.774845 | 4.981591 |
| $(1, 5)$ | 5.571996 | 5.375152 | 5.503769 | 5.611673 | 5.49763 | 5.478124 | 5.360295 | 4.796829 | 5.204838 | 6.113808 | 5.327918 |
| $(2, 1)$ | 4.23127 | 3.981119 | 4.089776 | 4.318423 | 4.123428 | 4.065036 | 3.814171 | 3.204795 | 3.586624 | 4.933363 | 3.913676 |
| $(2, 2)$ | 5.333136 | 5.150891 | 5.255156 | 5.388885 | 5.252282 | 5.23017 | 4.916448 | 4.180868 | 4.653807 | 6.113481 | 5.113902 |
| $(2, 3)$ | 5.952078 | 5.790924 | 5.892875 | 5.996593 | 5.88022 | 5.868162 | 5.581241 | 4.865612 | 5.331427 | 6.753958 | 5.762081 |
| $(2, 4)$ | 6.384036 | 6.233065 | 6.333797 | 6.42287 | 6.316605 | 6.309256 | 6.050293 | 5.382479 | 5.818485 | 7.195995 | 6.207994 |
| $(2, 5)$ | 6.716087 | 6.571108 | 6.671096 | 6.7515 | 6.651286 | 6.646667 | 6.4103 | 5.792401 | 6.194947 | 7.533891 | 6.548212 |
| $(3, 1)$ | 4.884026 | 4.679791 | 4.786094 | 4.950739 | 4.794069 | 4.760964 | 4.449398 | 3.741404 | 4.191345 | 5.640782 | 4.63375 |
| $(3, 2)$ | 5.976816 | 5.837207 | 5.921859 | 6.01931 | 5.913217 | 5.900912 | 5.570899 | 4.809556 | 5.295423 | 6.818207 | 5.812168 |
| $(3, 3)$ | 6.592743 | 6.473639 | 6.550942 | 6.626458 | 6.53811 | 6.5316 | 6.237857 | 5.53104 | 5.982665 | 7.457994 | 6.454351 |
| $(3, 4)$ | 7.023212 | 6.914075 | 6.987761 | 7.052468 | 6.973021 | 6.969231 | 6.706099 | 6.063726 | 6.472195 | 7.899718 | 6.897469 |
| $(3, 5)$ | 7.354377 | 7.251125 | 7.322664 | 7.380942 | 7.306833 | 7.304623 | 7.064743 | 6.48036 | 6.848804 | 8.237434 | 7.236069 |
| $(4, 1)$ | 5.333136 | 5.150891 | 5.255156 | 5.388885 | 5.252282 | 5.23017 | 4.916448 | 4.180868 | 4.653807 | 6.113481 | 5.113902 |
| $(4, 2)$ | 6.421372 | 6.302701 | 6.377463 | 6.456603 | 6.366763 | 6.358725 | 6.043974 | 5.303236 | 5.774893 | 7.289808 | 6.282868 |
| $(4, 3)$ | 7.035816 | 6.937512 | 7.002577 | 7.063513 | 6.990214 | 6.986085 | 6.709522 | 6.041408 | 6.464283 | 7.929322 | 6.92244 |
| $(4, 4)$ | 7.465554 | 7.377181 | 7.437502 | 7.489411 | 7.424429 | 7.422134 | 7.175741 | 6.578621 | 6.952893 | 8.370924 | 7.364337 |
| $(4, 5)$ | 7.796285 | 7.713783 | 7.771296 | 7.81782 | 7.757832 | 7.756601 | 7.532594 | 6.995257 | 7.327924 | 8.708572 | 7.70223 |
| $(5, 1)$ | 5.675471 | 5.505994 | 5.608894 | 5.724503 | 5.600033 | 5.58406 | 5.282262 | 4.549782 | 5.024368 | 6.468966 | 5.473993 |
| $(5, 2)$ | 6.760999 | 6.654626 | 6.723539 | 6.791736 | 6.7118 | 6.706147 | 6.411093 | 5.70515 | 6.153534 | 7.644699 | 6.6377 |
| $(5, 3)$ | 7.374565 | 7.288519 | 7.346379 | 7.398515 | 7.334416 | 7.331611 | 7.074456 | 6.450336 | 6.842341 | 8.28407 | 7.275813 |
| $(5, 4)$ | 7.803872 | 7.727754 | 7.780216 | 7.824352 | 7.768229 | 7.766756 | 7.538644 | 6.987335 | 7.329068 | 8.725608 | 7.717028 |
| $(5, 5)$ | 8.134347 | 8.064103 | 8.113373 | 8.152726 | 8.101394 | 8.100694 | 7.893879 | 7.401549 | 7.702193 | 9.06322 | 8.054526 |

**Table S12:** Regression equations for PHOTH-G: correlation analysis between energy and topological descriptors at $k = 1$

| $TD$ | Equation | $r^2$ | adj($r^2$) | $S.E$ | $F$ |
|---|---|---|---|---|---|
| $M_1\Re M_1$ | $0.472(M_1\Re M_1) - 9.003$ | 0.999 | 0.999 | 3.335 | 19487.196 |
| $M_1\Re M_2$ | $0.854(M_1\Re M_2) - 24.987$ | 0.991 | 0.991 | 9.175 | 2554.165 |
| $M_1\Re F$ | $0.404(M_1\Re F) - 28.014$ | 0.987 | 0.987 | 11.036 | 1758.325 |
| $M_1\Re GA$ | $1.03(M_k\Re GA) + 4.679$ | 1.000 | 1.000 | 1.884 | 61073.368 |
| $M_1\Re BM$ | $0.304(M_1\Re BM) - 14.81$ | 0.997 | 0.997 | 5.405 | 7402.159 |
| $M_1\Re TM$ | $0.274(M_1\Re TM) - 27.055$ | 0.998 | 0.988 | 10.435 | 1969.363 |
| $M_1\Re HG$ | $1.107(M_1\Re HG) + 18.142$ | 0.995 | 0.995 | 6.817 | 4645.274 |
| $M_1\Re HTM$ | $3.408(M_1\Re HTM) + 22.586$ | 0.992 | 0.992 | 8.53 | 2958.157 |
| $M_1\Re HBM$ | $3.356(M_1\Re HBM) + 19.948$ | 0.994 | 0.994 | 7.502 | 3832.053 |
| $M_1\Re BMG$ | $0.331(M_1\Re BMG) - 0.845$ | 1.000 | 1.000 | 0.331 | 1976067.07 |
| $M_1\Re BMH$ | $0.262(M_1\Re BMH) - 34.313$ | 0.982 | 0.981 | 13.041 | 1252.527 |

**Table S13:** Regression equations for S-G: correlation analysis between energy and topological descriptors at $k = 1$

| $TD$ | Equation | $r^2$ | adj($r^2$) | $S.E$ | $F$ |
|---|---|---|---|---|---|
| $M_1\Re M_1$ | $0.464(M_1\Re M_1) - 6.946$ | 0.999 | 0.999 | 2.049 | 31865.484 |
| $M_1\Re M_2$ | $0.843(M_1\Re M_2) - 18.795$ | 0.994 | 0.993 | 6.071 | 3607.884 |
| $M_1\Re F$ | $0.403(M_1\Re F) - 21.678$ | 0.993 | 0.993 | 6.527 | 3117.913 |
| $M_1\Re GA$ | $1.007(M_1\Re GA) + 3.142$ | 1.000 | 1.000 | 0.845 | 187531.648 |
| $M_1\Re BM$ | $0.3(M_1\Re BM) - 11.228$ | 0.998 | 0.998 | 3.468 | 11101.962 |
| $M_1\Re TM$ | $0.273(M_k\Re TM) - 20.749$ | 0.993 | 0.993 | 6.368 | 3277.48 |
| $M_1\Re HG$ | $1.079(M_1\Re HG) + 12.746$ | 0.998 | 0.998 | 3.484 | 11003.364 |
| $M_k\Re HTM$ | $3.315(M_1\Re HTM) + 15.836$ | 0.997 | 0.997 | 4.256 | 7363.391 |
| $M_k\Re HBM$ | $3.268(M_1\Re HBM) + 14.008$ | 0.998 | 0.997 | 3.806 | 9123.065 |
| $M_k\Re BMG$ | $0.325(M_1\Re BMG) - 0.897$ | 1.000 | 1.000 | 0.271 | 1827006.06 |
| $M_k\Re BMH$ | $0.26(M_1\Re BMH) - 26.058$ | 0.998 | 0.987 | 8.471 | 1841.741 |

**Table S14:** Regression equations for PH-G: correlation analysis between energy and topological descriptors at $k = 1$

| $TD$ | Equation | $r^2$ | adj($r^2$) | $S.E$ | $F$ |
|---|---|---|---|---|---|
| $M_1\Re M_1$ | $0.482(M_1\Re M_1) - 7.62$ | 0.999 | 0.999 | 2.453 | 23954.695 |
| $M_1\Re M_2$ | $0.897(M_1\Re M_2) - 19.485$ | 0.996 | 0.996 | 4.928 | 5919.294 |
| $M_1\Re F$ | $0.419(M_1\Re F) - 22.888$ | 0.992 | 0.992 | 7.087 | 2850.026 |
| $M_1\Re GA$ | $1.049(M_1\Re GA) + 3.021$ | 1.000 | 1.000 | 0.881 | 186052.744 |
| $M_1\Re BM$ | $0.314(M_1\Re BM) - 11.795$ | 0.998 | 0.998 | 3.316 | 13102.156 |
| $M_1\Re TM$ | $0.285(M_1\Re TM) - 21.826$ | 0.993 | 0.993 | 6.399 | 3500.722 |
| $M_1\Re HG$ | $1.13(M_1\Re HG) + 13.472$ | 0.997 | 0.997 | 4.036 | 8834.807 |
| $M_1\Re HTM$ | $3.491(M_1\Re HTM) + 17.34$ | 0.995 | 0.995 | 5.437 | 4859.001 |
| $M_1\Re HBM$ | $3.428(M_1\Re HBM) + 14.991$ | 0.997 | 0.997 | 4.565 | 6903.084 |
| $M_1\Re BMG$ | $0.337(M_1\Re BMG) - 1.382$ | 1.000 | 1.000 | 0.716 | 281643.023 |
| $M_1\Re BMH$ | $0.279(M_1\Re BMH) - 27.353$ | 0.992 | 0.992 | 7.087 | 2850.026 |

**Table S15:** Regression equations for PHOTH-G$(m, n)$ correlating energy and the topological descriptors when $k = 2$

| $TD$ | Equation | $r^2$ | adj($r^2$) | $S.E$ | $F$ |
|---|---|---|---|---|---|
| $M_2\Re M_1$ | $0.245(M_2\Re M_1) - 2.886$ | 1.000 | 1.000 | 1.022 | 207818.44 |
| $M_2\Re M_2$ | $0.235(M_2\Re M_2) - 10.642$ | 0.998 | 0.998 | 3.819 | 14852.877 |
| $M_2\Re F$ | $0.116(M_2\Re F) - 11.829$ | 0.998 | 0.998 | 4.43 | 11032.226 |
| $M_2\Re GA$ | $1.025(M_2\Re GA) + 4.183$ | 1.000 | 1.000 | 1.659 | 78771.323 |
| $M_2\Re BM$ | $0.12(M_2\Re BM) - 6.881$ | 0.999 | 0.999 | 2.446 | 36234.758 |
| $M_2\Re TM$ | $0.077(M_2\Re TM) - 11.439$ | 0.998 | 0.998 | 4.228 | 12114.847 |
| $M_2\Re HG$ | $4.315(M_2\Re HG) + 13.451$ | 0.997 | 0.997 | 5.053 | 8474.344 |
| $M_2\Re HTM$ | $26.445(M_2\Re HTM) + 17.154$ | 0.996 | 0.995 | 6.456 | 5181.833 |
| $M_2\Re HBM$ | $17.443(M_2\Re HBM) + 15.364$ | 0.996 | 0.996 | 5.765 | 6506.383 |
| $M_2\Re BMG$ | $0.251(M_2\Re BMG) + 0.452$ | 1.000 | 1.000 | 0.327 | 2030815.8 |
| $M_2\Re BMH$ | $0.057(M_2\Re BMH) - 16.144$ | 0.996 | 0.996 | 5.912 | 6183.732 |

**Table S16:** Regression equations for S-G$(m,n)$ correlating energy and the topological descriptors when $k=2$

| $TD$ | Equation | $r^2$ | adj$(r^2)$ | $S.E$ | $F$ |
|---|---|---|---|---|---|
| $M_2\Re M_1$ | $0.24(M_2\Re M_1) - 0.52$ | 0.984 | 0.983 | 9.798 | 1371.001 |
| $M_2\Re M_2$ | $0.230(M_2\Re M_2) - 5.976$ | 0.984 | 0.984 | 9.511 | 1456.451 |
| $M_2\Re F$ | $0.114(M_2\Re F) - 6.986$ | 0.985 | 0.984 | 9.444 | 1477.269 |
| $M_2\Re GA$ | $0.998(M_2\Re GA) + 5.313$ | 0.982 | 0.981 | 10.342 | 1228.184 |
| $M_2\Re BM$ | $0.117(M_2\Re BM) - 3.093$ | 0.984 | 0.983 | 9.607 | 1427.036 |
| $M_2\Re TM$ | $0.076(M_2\Re TM) - 6.653$ | 0.985 | 0.984 | 9.465 | 1470.678 |
| $M_2\Re HG$ | $4.182(M_2\Re HG) + 12.203$ | 0.978 | 0.977 | 11.303 | 1024.376 |
| $M_2\Re HTM$ | $25.568(M_2\Re HTM) + 14.912$ | 0.976 | 0.975 | 11.741 | 947.844 |
| $M_2\Re HBM$ | $16.886(M_2\Re HBM) + 13.607$ | 0.977 | 0.976 | 11.527 | 984.106 |
| $M_2\Re BMG$ | $0.244(M_2\Re BMG) + 2.492$ | 0.983 | 0.982 | 10.029 | 1307.414 |
| $M_2\Re BMH$ | $0.056(M_2\Re BMH) - 10.26$ | 0.984 | 0.984 | 9.515 | 1455.118 |

**Table S17:** Regression equations for PH-G$(m,n)$ correlating energy and the topological descriptors when $k=2$

| $TD$ | Equation | $r^2$ | adj$(r^2)$ | $S.E$ | $F$ |
|---|---|---|---|---|---|
| $M_2\Re M_1$ | $0.25(M_2\Re M_1) - 2.905$ | 1.000 | 1.000 | 1.098 | 119580.826 |
| $M_2\Re M_2$ | $0.241(M_2\Re M_2) - 8.585$ | 0.999 | 0.999 | 2.453 | 23954.695 |
| $M_2\Re F$ | $0.118(M_2\Re F) - 9.826$ | 0.998 | 0.998 | 3.104 | 14957.076 |
| $M_2\Re GA$ | $1.043(M_2\Re GA) + 2.523$ | 1.000 | 1.000 | 0.673 | 318525.039 |
| $M_2\Re BM$ | $0.123(M_2\Re BM) - 5.806$ | 0.999 | 0.999 | 1.782 | 45399.43 |
| $M_2\Re TM$ | $0.079(M_2\Re TM) - 9.419$ | 0.999 | 0.999 | 2.889 | 17263.493 |
| $M_2\Re HG$ | $4.39(M_2\Re HG) + 9.635$ | 0.999 | 0.999 | 2.748 | 19092.074 |
| $M_2\Re HTM$ | $26.965(M_2\Re HTM) + 12.691$ | 0.998 | 0.998 | 3.79 | 10022.991 |
| $M_2\Re HBM$ | $17.76(M_2\Re HBM) + 11.165$ | 0.998 | 0.998 | 3.244 | 13689.605 |
| $M_2\Re BMG$ | $0.255(M_2\Re BMG) - 0.356$ | 1.000 | 1.000 | 0.455 | 697499.183 |
| $M_2\Re BMH$ | $0.058(M_2\Re BMH) - 12.819$ | 0.998 | 0.998 | 3.577 | 11251.93 |

**Table S18:** Regression equations for PHOTH-G$(m,n)$ correlating energy and the topological descriptors when $k=3$

| $TD$ | Equation | $r^2$ | adj$(r^2)$ | $S.E$ | $F$ |
|---|---|---|---|---|---|
| $M_3\Re M_1$ | $0.179(M_3\Re M_1)+15.075$ | 0.997 | 0.997 | 5.290 | 7729.592 |
| $M_3\Re M_2$ | $0.125(M_3\Re M_2)+23.366$ | 0.992 | 0.992 | 8.424 | 3033.993 |
| $M_3\Re F$ | $0.061(M_3\Re F)+19.474$ | 0.995 | 0.995 | 6.928 | 4496.191 |
| $M_3\Re GA$ | $1.039(M_3\Re GA)+5.727$ | 0.999 | 0.999 | 2.362 | 38865.084 |
| $M_3\Re BM$ | $0.074(M_3\Re BM)+19.936$ | 0.995 | 0.994 | 7.134 | 4239.668 |
| $M_3\Re TM$ | $0.041(M_3\Re TM)+20.74$ | 0.994 | 0.994 | 7.411 | 3927.208 |
| $M_3\Re HG$ | $6.576(M_3\Re HG)-52.476$ | 0.965 | 0.963 | 18.251 | 628.297 |
| $M_3\Re HTM$ | $40.568(M_3\Re HTM)-93.298$ | 0.869 | 0.863 | 35.211 | 151.979 |
| $M_3\Re HBM$ | $27.674(M_3\Re HBM)-71.585$ | 0.929 | 0.926 | 25.912 | 300.088 |
| $M_3\Re BMG$ | $0.211(M_3\Re BMG)+10.305$ | 0.999 | 0.999 | 3.639 | 16355.639 |
| $M_3\Re BMH$ | $0.025(M_3\Re BMH)+24.455$ | 0.992 | 0.991 | 8.871 | 2733.476 |

**Table S19:** Regression equations for S-G$(m,n)$ correlating energy and the topological descriptors when $k=3$

| $TD$ | Equation | $r^2$ | adj$(r^2)$ | $S.E$ | $F$ |
|---|---|---|---|---|---|
| $M_3\Re M_1$ | $0.176(M_3\Re M_1)+9.962$ | 0.999 | 0.999 | 2.826 | 16734.084 |
| $M_3\Re M_2$ | $0.122(M_3\Re M_2)+15.331$ | 0.997 | 0.997 | 4.153 | 7735.707 |
| $M_3\Re F$ | $0.6(M_3\Re F)+12.634$ | 0.998 | 0.998 | 3.576 | 10438.953 |
| $M_3\Re GA$ | $1.015(M_3\Re GA)+3.912$ | 1.000 | 1.000 | 1.023 | 127762.359 |
| $M_3\Re BM$ | $0.072(M_3\Re BM)+13.122$ | 0.998 | 0.998 | 3.604 | 10282.242 |
| $M_3\Re TM$ | $0.04(M_3\Re TM)+13.517$ | 0.998 | 0.997 | 3.76 | 9442.792 |
| $M_3\Re HG$ | $6.367(M_3\Re HG)-36.208$ | 0.969 | 0.967 | 13.47 | 714.567 |
| $M_3\Re HTM$ | $36.826(M_3\Re HTM)-57.1$ | 0.857 | 0.851 | 28.82 | 138.12 |
| $M_3\Re HBM$ | $26.436(M_3\Re HBM)-48.262$ | 0.933 | 0.93 | 19.805 | 318.174 |
| $M_3\Re BMG$ | $0.206(M_3\Re BMG)+6.81$ | 0.999 | 0.999 | 1.942 | 35466.165 |
| $M_3\Re BMH$ | $0.025(M_3\Re BMH)+15.905$ | 0.997 | 0.997 | 4.311 | 7176.005 |

**Table S20:** Regression equations for PH-G$(m, n)$ correlating energy and the topological descriptors when $k = 3$

| $TD$ | Equation | $r^2$ | adj$(r^2)$ | $S.E$ | $F$ |
|---|---|---|---|---|---|
| $M_3\Re M_1$ | $0.183(M_3\Re M_1) + 9.911$ | 0.999 | 0.999 | 2.655 | 20443.302 |
| $M_3\Re M_2$ | $0.128(M_3\Re M_2) + 16.533$ | 0.996 | 0.996 | 5.069 | 5592.464 |
| $M_3\Re F$ | $0.062(M_3\Re F) + 12.683$ | 0.998 | 0.998 | 3.430 | 12244.57 |
| $M_3\Re GA$ | $1.062(M_3\Re GA) + 4.08$ | 1.000 | 1.000 | 1.346 | 79601.748 |
| $M_3\Re BM$ | $0.075(M_3\Re BM) + 13.779$ | 0.997 | 0.997 | 4.071 | 8684.606 |
| $M_3\Re TM$ | $0.042(M_3\Re TM) + 13.927$ | 0.997 | 0.997 | 3.963 | 9166.628 |
| $M_3\Re HG$ | $7.436(M_3\Re HG) - 40.006$ | 0.991 | 0.991 | 7.478 | 2557.605 |
| $M_3\Re HTM$ | $62.56(M_3\Re HTM) - 97.338$ | 0.986 | 0.985 | 9.383 | 1616.01 |
| $M_3\Re HBM$ | $34.324(M_3\Re HBM) - 60.321$ | 0.985 | 0.984 | 9.72 | 1504.387 |
| $M_3\Re BMG$ | $0.214(M_3\Re BMG) + 6.613$ | 1.000 | 1.000 | 1.713 | 49130.303 |
| $M_3\Re BMH$ | $0.026(M_3\Re BMH) + 17.144$ | 0.996 | 0.995 | 5.204 | 5232.43 |
